# Serotonin and brain function: a tale of two receptors

**DOI:** 10.1177/0269881117725915

**Published:** 2017-08-31

**Authors:** RL Carhart-Harris, DJ Nutt

**Affiliations:** Psychedelic Research Group, Neuropsychopharmacology Unit, Centre for Psychiatry, Division of Brain Sciences, Department of Medicine, Imperial College London, London, UK

**Keywords:** Depression, serotonin, psychedelics

## Abstract

Previous attempts to identify a unified theory of brain serotonin function have largely failed to achieve consensus. In this present synthesis, we integrate previous perspectives with new and older data to create a novel bipartite model centred on the view that serotonin neurotransmission enhances two distinct adaptive responses to adversity, mediated in large part by its two most prevalent and researched brain receptors: the 5-HT1A and 5-HT2A receptors. We propose that *passive coping* (i.e. tolerating a source of stress) is mediated by postsynaptic 5-HT1AR signalling and characterised by stress moderation. Conversely, we argue that *active coping* (i.e. actively addressing a source of stress) is mediated by 5-HT2AR signalling and characterised by enhanced plasticity (defined as capacity for change). We propose that 5-HT1AR-mediated stress moderation may be the brain’s default response to adversity but that an improved ability to change one’s situation and/or relationship to it via 5-HT2AR-mediated plasticity may also be important – and increasingly so as the level of adversity reaches a critical point. We propose that the 5-HT1AR pathway is enhanced by conventional 5-HT reuptake blocking antidepressants such as the selective serotonin reuptake inhibitors (SSRIs), whereas the 5-HT2AR pathway is enhanced by 5-HT2AR-agonist psychedelics. This bipartite model purports to explain how different drugs (SSRIs and psychedelics) that modulate the serotonergic system in different ways, can achieve complementary adaptive and potentially therapeutic outcomes.

## Introduction

### Overview

The aim of this paper is to discuss the function of brain serotonin (5-HT) transmission by focusing on two of its major receptor subtypes, the 5-HT1AR and 5-HT2AR. Our selective focus on these receptors is justified by their dense and widespread expression in the human brain (Beliveau et al., 2016), diametrically opposite functional effects (Araneda and Andrade, 1991) and extensive evidence implicating both in psychiatric disorders and their treatment (Chattopadhyay, 2007). We believe that a fuller understanding of the function of 5-HT1A and particularly, 5-HT2A receptor signalling motivates a revision of current thinking on a well-known problem in neuropsychopharmacology, namely: what principal function is served by brain serotonin transmission? Broadly consistent with prior theories (Deakin, 2013), we maintain that a key function of brain 5-HT is to moderate anxiety and stress, and promote patience and coping (Miyazaki et al., 2012) via (postsynaptic) 5-HT1AR signalling. Crucially however, we also extend on this by proposing that a second major function of brain 5-HT is to open a window of plasticity for greater adaptation (Branchi, 2011), mediated in large part by 5-HT2AR signalling. This bipartite model is consistent with a ‘flexible coping’ model of brain serotonin function, in which postsynaptic 5-HT1ARs mediate so-called ‘passive coping’ (i.e. tolerating but not necessarily dealing with a source of psychological pain) and 5-HT2ARs mediate ‘active coping’ (actively dealing with a source of psychological pain by changing one’s relationship to it) (Puglisi-Allegra and Andolina, 2015). Note: we use the term ‘plasticity’ in a broad sense throughout this paper to refer to the *capacity for change* and we address our intentional neglect of the other serotonin receptors in the discussion section as well as immediately below.

The charge that our neglect of the functioning of the full range of serotonin receptors means that the present paper cannot be considered a fully comprehensive model of brain serotonin function is one we accept. However, we propose that the functioning of signalling at other serotonin receptors (than 1A and 2A) may, in several cases, be comfortably incorporated into either (or both) arms of the bipartite model we introduce below – and we encourage attempts to do this. A final introductory caveat is that signalling at serotonin receptors can have more than one function, depending on such factors as: basal serotonin efflux and related synaptic concentrations, the specific localisation of the relevant receptor subtype (e.g. whether they are pre- or postsynaptic), the temporal development or time course of a specific pharmacological manipulation, and the animal’s present behavioural state (e.g. see Mitchell, 2005 for a relevant review). As much as is possible, we have endeavoured to acknowledge such inherent complexities in the serotonin system – particularly when we feel they are critical for a proper comprehension of the relevant phenomenon – but this has had to be balanced against considerations of parsimony and focus – in any already extensive narrative review.

With these caveats entered, let us return to the main focus of this paper: brain serotonin functioning – as seen through postsynaptic 5-HT1A and 5-HT2A receptor signalling. The 5-HT1AR is highly expressed in brain regions involved in regulating stress and emotion and 5-HT has an especially high affinity for its 1A receptor (Peroutka and Snyder, 1979). We suggest that the 5-HT1AR and its associated functions dominate 5-HT transmission under normal conditions but that 5-HT2AR signalling also serves a role that becomes increasingly important during extreme states when 5-HT release is elevated. We propose that 5-HT mediates stress moderation and plasticity-mediated adaptability in response to different levels of stress and adversity, via its postsynaptic 1A and 2A receptors respectively. We acknowledge that agonism at other 5-HT receptors has also been linked with neurotrophic factors and other molecular markers of neuroplasticity (Kraus et al., 2017); however, our focus here is on the remarkable psychological and functional plasticity associated with the acute ‘psychedelic’ state – as produced by psychedelic drugs such as LSD and psilocybin (Carhart-Harris et al., 2016c) – and the enduring changes that appear to follow from exposure to these drugs’ effects (e.g. MacLean et al., 2011). We also propose that combined signalling at the 5-HT1A and 2A receptors has a generally complementary influence on mood, facilitating stress relief (5-HT1AR-mediated) but also a flexibility of mind (5-HT2AR-mediated) that under favourable conditions (Alboni et al., 2017; Branchi, 2011; Chiarotti et al., 2017; Hartogsohn, 2016), is conducive to positive mood (Hirt et al., 2008; Schmid et al., 2015). In what follows, we present evidence supporting these hypotheses and discuss their clinical significance.

### The function of brain serotonin is an enigma

There have been several attempts to identify a unifying function of dopaminergic transmission in the brain (Berridge and Robinson, 1998; Schultz, 2010; Schwartenbeck et al., 2014) and similar attempts have been made for serotonin (Andrews et al., 2015; Azmitia, 2007; Branchi, 2011; Dayan and Huys, 2009; Deakin, 1998). Most researchers acknowledge that the function of the 5-HT system remains ‘elusive’ (Dayan and Huys, 2009) and ‘a puzzle’ (Cools et al., 2008; Dayan and Huys, 2015; Seymour et al., 2012) and it is argued here that this may be due to the special diversity and complexity of the serotonin system with its many receptor subtypes (Hoyer et al., 1994), extensive innervation of the brain and paracrine style of transmission (Hornung, 2003; Jennings, 2013). The notion that 5-HT is an enigma among neuromodulators (said to be ‘involved in everything but responsible for nothing’ (Muller and Homberg, 2015)) is relevant here, and it is argued that the riddle of 5-HT can only be solved by focusing on its individual receptor subtypes.

Accordingly, given the inherent complexity of the serotonin system, one strategy for understanding its functioning is to focus on a select number of receptor subtypes that have been particularly well characterised. From this foundation, one might then consider whether other serotonin receptor subtypes can be incorporated into the associated model, or whether one or more additional models are required to cover the full range of functions associated with brain serotonin transmission. Following this approach, we have chosen to concentrate on the 5-HT1A and 5-HT2A receptors. Our reasons for doing this are (at least) three-fold, and include: (1) the prevalence of their expression in the human brain and specific localisation – e.g. in stress circuitry (5-HT1AR) and high-level cortex (5-HT2AR) (e.g. Beliveau et al., 2016); (2) compelling evidence for their involvement in the pharmacology of different psychiatric disorders and medications (Celada et al., 2004); and (3) their apparent functional pre-eminence and opposition – as has been noted by others (Azmitia, 2001). Following on from this last point, the 5-HT1A and 5-HT2A receptors show diametrically opposite responses to their endogenous ligand, with 5-HT1A receptor signalling being inhibitory and 5-HT2A receptor signalling being excitatory (Araneda and Andrade, 1991; Azmitia, 2001; Charig et al., 1986; Fletcher et al., 2007). This stark functional opposition is intriguing – and motivates us to ask *why* this should be the case, and what purpose it serves? We suggest that inherent diversity within the serotonergic system relates to its capacity for flexibly and adaptably responding to different degrees of adversity and challenge in the organism’s environment, with distinct responses mediated by distinct serotonergic pathways.

As noted above, an obvious caveat here is that 5-HT receptors we do not specifically focus on in the present review may complement one or the other of these two pathways – and may also modulate unrelated physiological and behavioural functions. For example, signalling at 5-HT receptors other than the 2A receptor has been associated with neuroplasticity (Kraus et al., 2017) – and thus, may also feed into pathway 2 (below). Similarly blockade of certain 5-HT receptors (e.g. 5-HT2C, 5-HT7 and even 5-HT2A) may complement pathway 1 (below). However, a thorough coverage of this matter is beyond the scope of this article.

In what follows, focus is directed to 5-HT1A and 5-HT2A receptor signalling and research pertaining to their associated functions. It is argued that studying potent serotonergic compounds such as rapid-acting, highly effective 5-HT releasers (such as 3,4-methylenedioxymethamphetanine, MDMA (Baumann et al., 2008; Heifets and Malenka, 2016)) and direct 5-HT2AR agonist psychedelic drugs such as psilocybin and lysergic acid diethylamide, LSD (Glennon et al., 1984; Vollenweider et al., 1998), can be particularly informative about the function of serotonergic transmission in the brain because their acute and longer-term effects are especially marked and novel (Griffiths et al., 2008; Mithoefer et al., 2013), and there is a growing literature on human research with such drugs, including an increasing number of neuroimaging studies (Carhart-Harris et al., 2013b, 2015b; Muthukumaraswamy et al., 2013) and clinical trials (Bogenschutz et al., 2015; Carhart-Harris et al., 2016a; Gasser et al., 2014; Griffiths et al., 2016; Grob et al., 2011; Mithoefer et al., 2011; Ross et al., 2016; Sanches et al., 2016) – see Carhart-Harris and Goodwin (2017) for a review.

Note: we acknowledge that MDMA also releases dopamine (DA) and noradrenaline (NA) (Baumann et al., 2008) but its 5-HT releasing properties are many times greater than its catecholamine releasing properties, e.g. 5-HT release in the frontal cortex is approximately 5 times that of DA release (Golembiowska et al., 2016), preferential 5-HT versus DA and NA release is unusual for an amphetamine, and MDMA’s subjective effects are also distinct from those of other more conventional amphetamines (Bedi et al., 2014).

## Serotonin receptor subtypes

### What is the 5-HT2AR and where is it expressed?

The 5-HT2AR is one of at least 14 different 5-HT receptor subtypes expressed in the mammalian brain (Glennon, 2000), and like almost all of these, it is a G protein-coupled receptor (GPCR). In the context of neurotransmission, the principal effect of 5-HT binding to the 5-HT2AR is to increase the excitability of the host neuron, and the 5-HT2AR is the main excitatory GPCR of the serotonin receptor family (Andrade, 2011).

**Figure 1.**
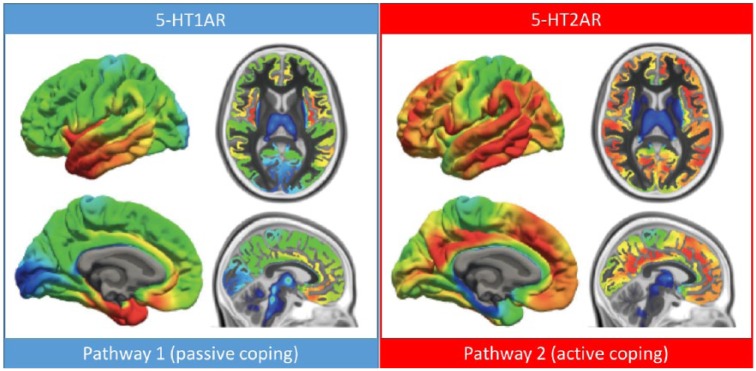
Regional distribution of serotonin 1A (left) and 2A receptors (right) in healthy volunteers as measured using PET imaging and radioligands selective for the 5-HT 1A and 2A receptors. Pathway 1 refers to the ‘passive coping’ pathway hypothesised to be mediated by 5-HT1AR signalling and concerned with passive endurance, and ‘pathway 2’ refers to the ‘active coping’ pathway hypothesised to be mediated by 5-HT2AR signalling and concerned with an active change in outlook and/or behaviour. Images reproduced from (Beliveau et al., 2016) with permission. Note: The dense expression of the 5-HT1AR in medial temporal lobe regions and particularly the hippocampus is not clearly evident in the relevant maps shown here (left) but can be seen in values presented in the paper itself, as well as others (Pazos and Palacios, 1985; Pazos et al., 1987).

The 5-HT2AR is predominantly a cortical receptor; indeed, it is the most abundant 5-HT receptor in the cortex (Varnas et al., 2004). In humans, the density of 5-HT2AR expression is relatively high throughout the cortex and especially so in high-level associative cortex – such as regions belonging to the so-called default-mode network (see Figure 1) (Beliveau et al., 2016). 5-HT2AR expression is considerably higher in the cortex than in subcortical structures such as the thalamus, basal ganglia, and hippocampus (Gross-Isseroff et al., 1990; Hall et al., 2000) – with minimal/negligible expression in the cerebellum and brainstem (Hall et al., 2000). The predominantly cortical expression of the 5-HT2AR places it at a high evolutionary and hierarchical level and as we will discuss later (e.g. Section 4.4), this is likely to have important functional implications.

In terms of its cellular and laminar localisation, 5-HT2A receptors are most densely expressed on the dendrites of excitatory glutamatergic pyramidal neurons, particular in layer V of the cortex (Weber and Andrade, 2010). One study found that almost all glutamatergic neurons in layers II-V of the monkey and human prefrontal cortex (PFC) expressed 5-HT2ARs, whereas only about 30% of GABAergic interneurons within the same layers exhibited 5-HT2AR expression (de Almeida and Mengod, 2007). Thus, cortical pyramidal neurons are likely to be especially sensitive to modulation via 5-HT activating 5-HT2ARs, and furthermore, the laminar localisation of 5-HT2ARs (e.g. in layer V of the cortex) corresponds well with the localisation of axon terminals of serotonergic neurons, particularly in the cortex (Blue et al., 1988). These data imply that cortical 5-HT2ARs should be sensitive to changes in synaptic serotonin concentrations (Tyacke and Nutt, 2015). A well-demonstrated effect of (prefrontal) cortical 5-HT2AR signalling is the initiation of a negative feedback mechanism which inhibits the firing of serotonergic neurons in the dorsal raphe nucleus (Boothman et al., 2003; Quesseveur et al., 2013), suggesting that the 5-HT2AR plays a crucial role in regulating the release of serotonin in the cortex, via a top-down modulatory influence on a cortical-raphe inhibitory feedback circuit (Sharp et al., 2007; Vazquez-Borsetti et al., 2009).

### What is the 5-HT1AR and where is it expressed?

Identified in the early 1980s as a distinct 5-HT receptor subtype (Pedigo et al., 1981), the 5-HT1AR is densely expressed in midbrain, limbic and cortical regions (Varnas et al., 2004). 5-HT1AR agonism causes host-cell hyperpolarisation and an inhibition of firing via G protein-mediated mechanisms (Oleskevich et al., 2005). The 5-HT1AR is highly expressed on serotonergic neurons in the dorsal and median raphe nuclei where it functions as a presynaptic autoreceptor – exerting a strong homeostatic control over 5-HT neuron firing rates and thus, 5-HT efflux in the forebrain (Lanfumey and Hamon, 2000). The majority of 5-HT1A receptors are expressed postsynaptically in many brain regions, particularly the limbic system (especially the hippocampus) and cortex (Pazos et al., 1987; Varnas et al., 2004) see Figure 1. Presynaptic 5-HT1ARs readily desensitise following exposure to increased 5-HT availability (e.g. through chronic **selective serotonin reuptake inhibitors** (SSRIs)) but postsynaptic 5-HT1ARs do not (Lanfumey and Hamon, 2000), although they do appear to downregulate in response to stress (Berton et al., 1998; Lopez et al., 1999) – and perhaps relatedly, to electroconvulsive shock (Burnet et al., 1955, 1999). In summary, based on its high density of expression, localisation to regions densely innervated by serotonergic projections (such as the hippocampus) and high affinity for its endogenous ligand, the postsynaptic 5-HT1AR is serotonin’s principal inhibitory receptor in the brain.

### Serotonin 2A versus 1A receptor signalling

At a basic level, the principal effect of 5-HT2AR activation is to increase the excitability of the host neuron (Andrade, 2011). If the host neuron is excitatory (e.g. a pyramidal neuron), the outcome of 5-HT2AR stimulation may be to increase its firing and the firing of those cells that it projects to. If the host cell is inhibitory (e.g. a GABAergic interneuron), the net result of 5-HT2AR stimulation will be to increase its firing and so enhance its inhibitory influence onto the neurons to which it projects (Andrade, 2011). Given that 5-HT2ARs are expressed mostly on excitatory neurons (at least in the cortex – where their expression is highest) one might expect release of endogenous 5-HT in the cortex to elicit a mostly excitatory effect but this is not what is typically observed (Hajos et al., 2003; Jacobs and Azmitia, 1992; Puig et al., 2005). For example, *in vivo* studies investigating the effect of dorsal raphe nucleus stimulation (inducing an increase in cortical 5-HT efflux) on cellular activity in the medial PFC (mPFC) have observed a decrease in the firing rate of the majority of pyramidal cells recorded (Hajos et al., 2003; Puig et al., 2005). Importantly, this effect appears to be modulated via postsynaptic 5-HT1ARs, since it could be prevented by a selective 5-HT1AR antagonist (Hajos et al., 2003; Puig et al., 2005). Consistently, chronic dorsal raphe stimulation was found to decrease metabolism in limbic regions, alongside decreases in depressive behaviours, presumably via inhibitory postsynaptic 5-HT1ARs (Urban et al., 2016).

It is a well-replicated finding that postsynaptic 5-HT1AR and 5-HT2AR activation produces opposite effects on single cell activity, with 5-HT1AR signalling having a hyperpolarising (inhibitory) effect, and 5-HT2AR activation causing a depolarising (excitatory) effect (Andrade, 2011; Araneda and Andrade, 1991). Up to 80% of pyramidal neurons in the PFC co-express 5-HT1A and 5-HT2A receptors (Amargos-Bosch et al., 2004). Studies in the 1970s and 80s suggested that 5-HT has an appreciably higher affinity for its 1A than 2A receptor (Hoyer et al., 1985; Peroutka and Snyder, 1979) but further research with 5-HT2AR agonist ligands suggest that, like other neuromodulator receptors (Skinbjerg et al., 2012) the 5-HT2A receptor can exist in a low (G-protein uncoupled) or high affinity (G-protein coupled) state – and when in their high-affinity state, 5-HT has a higher affinity for its 5-HT2AR than previously appreciated (Sleight et al., 1996). Under normal conditions, 5-HT1AR signalling seems to dominate serotoninergic functioning in cortical as well as limbic regions (Puig et al., 2005). However, as we will discuss later (e.g. Section 4), the 5-HT2A receptor is still likely to be functionally relevant, and we predict, increasingly so during states of exceptionally high adversity (Amargos-Bosch et al., 2004; Puig et al., 2005). In this context, the possibility that high-affinity 5-HT2ARs upregulate (Benekareddy et al., 2010; Berton et al., 1998) and 5-HT1ARs downregulate during extreme adversity (Berton et al., 1998; Lopez et al., 1999) is an intriguing one, which seems deserving of further study.

The opposite effect of electroconvulsive shock on 5-HT1A and 5-HT2A receptor functioning in rats may be relevant here, with (hippocampal but not the dentate gyrus) 5-HT1AR expression appearing to decrease post ECS while 5-HT2AR functioning increases (Burnet et al., 1995, 1999). Conversely however, ECS/electroconvulsive therapy appears to downregulate 5-HT2AR binding in primates (Strome et al., 2005) and humans (Yathamet al., 2010) – an effect that is more consistent with that of conventional antidepressant medications (Yatham et al., 1999) as well as direct 5-HT2AR agonism (Buckholtz et al., 1990) – while also being the logical consequence of acutely enhanced 5-HT release with ECS/ECT (Zis et al., 1992).

## Psychological functions associated with brain 5-HT

### Impulsivity and aggression

One of the most reliable behavioural effects of reducing 5-HT transmission in the brain is to increase impulsive and aggressive behaviours (Audero et al., 2013; Brown et al., 1979; Duke et al., 2013; Mosienko et al., 2015; Soubrie, 1986). Indeed, some of the earliest hypotheses on the function of 5-HT in the brain proposed that it serves to suppress behavioural response to pain (Harvey et al., 1975), anxiety (Wise et al., 1970) and aversive stimuli more generally (Deakin and Graeff, 1991; Soubrie, 1986) and these ideas continue to have traction (Deakin, 2013; Yanowitch and Coccaro, 2011). The anti-aggression effects of 5-HT enhancing compounds led to them being called ‘serenics’ (Olivier and Moss, 1990), a fitting term in our view, and one that is also apt in relation to the subjective effects of MDMA, a particularly potent 5-HT releaser. Related to these hypotheses, is the notion that 5-HT transmission enables a person to better tolerate delay (Soubrie, 1986), and the patience-promoting properties of 5-HT have recently received significant experimental support (Fonseca et al., 2015; McDannald, 2015; Miyazaki et al., 2012, 2014; Ranade et al., 2014). Low concentrations of the serotonin metabolite (5-HIAA), implying low central 5-HT function, have been associated with impulsivity (Fairbanks et al., 2001), aggression (Brown and Linnoila, 1990) and suicidal behaviour (Asberg et al., 1976), and tryptophan depletion (a diet-based approach that produces a transient depletion of central 5-HT) has also been found to enhance impulsivity and aggression (Dougherty et al., 1999, 2010). In contrast, tryptophan supplementation (Duke et al., 2013), acute MDMA administration (Ramaekers and Kuypers, 2006; van Wel et al., 2012), acute fenfluramine (Cherek and Lane, 2001) and chronic 5-HT reuptake inhibitor administration (Butler et al., 2010; Wolff and Leander, 2002), all of which are known to increase central 5-HT function, have all been found to reduce impulsivity and aggression. For a more in-depth discussion of the complexities of the relationship between brain 5-HT and aggression, including some contradictory findings to the rule that low synaptic 5-HT is associated with increased aggression, see this review (Mitchell, 2005).

#### 5-HT1AR signalling, impulsivity and aggression

There are solid grounds to believe that the anti-aggression and impulsivity effects of 5-HT are mediated by postsynaptic 5-HT1A receptor signalling (Sanchez and Hyttel, 1994; Schreiber and De Vry, 1993), with some contribution from postsynaptic 5-HT1B receptors (Ramboz et al., 1996; Sijbesma et al., 1991). Assessing the functional effects of 5-HT1A receptor manipulations is complicated, however, owing to the opposing influences of pre- and postsynaptic 1A receptor activation. Prior to a time-dependent 5-HT1A autoreceptor desensitisation by reuptake blockers (Le Poul et al., 1995), stimulation of these presynaptic 5-HT1A receptors reduces serotonin efflux, whereas postsynaptic 5-HT1A receptor activation is an important (and often clinically desirable) consequence of increased serotonin efflux (Artigas, 2013b). Moreover, selective 5-HT1AR antagonists or full 5-HT1AR agonists are not available for human use (beyond the very low doses used in PET imaging), and so cannot be used to incisively inform on this matter. With these caveats, it can be relatively safely inferred that (postsynaptic) 5-HT1AR agonism appears to reduce aggressive and impulsive behaviours (de Boer and Koolhaas, 2005; Olivier et al., 1989; Popova et al., 2007; Sanchez and Hyttel, 1994; White et al., 1991; Wolff and Leander, 2002). Note, however, that many 5-HT1A receptor agonists are in fact, only partial agonists; thus, their impact on net 5-HT1AR signalling is dependent on basal 5-HT efflux and competition with the full agonist endogenous ligand, 5-HT itself (Mitchell, 2005).

It has been claimed that the 5-HT1AR is the most prevalent and well-distributed 5-HT receptor in the brain (Paterson et al., 2013; Varnas et al., 2004). Serotonin has a high affinity for this receptor subtype (Peroutka and Snyder, 1979), serotonergic projections densely innervate 5-HT1AR-rich regions (Hornung, 2003) and 5-HT concentrations may be higher in 5-HT1AR-rich subcortical/limbic regions than in the 5-HT2AR-rich cortex during basal conditions (Bose et al., 2011; Erritzoe et al., 2010; Kirby et al., 1995; Rueter and Jacobs, 1996), although see Adell et al. (1991) and Hjorth and Sharp (1991). These factors imply that manipulation of synaptic 5-HT concentrations will significantly impact on postsynaptic 5-HT1AR signalling and limbic functioning. With this in mind, it is telling that 5-HT lesions and depletion both tend to promote impulsivity and aggression (Audero et al., 2013; Dougherty et al., 1999), whereas stimulating serotonin function tends to reduce these behaviours (Miyazaki et al., 2014). It is also relevant that the potent 5-HT releaser, MDMA, has marked pro-social, pro-empathy, anti-aggressive effects during the acute phase (Bedi et al., 2010, 2014; Frye et al., 2014; Hysek et al., 2012, 2014a; Kamboj et al., 2015; Kirilly et al., 2006; Schmid et al., 2014; Stewart et al., 2014), perhaps via an inhibitory action on activity in limbic regions (Carhart-Harris et al., 2015b), and some of these effects in rodents’ can be attenuated by pre-treatment with a 5-HT1A receptor antagonist (Hunt et al., 2011).

**Figure 2.**
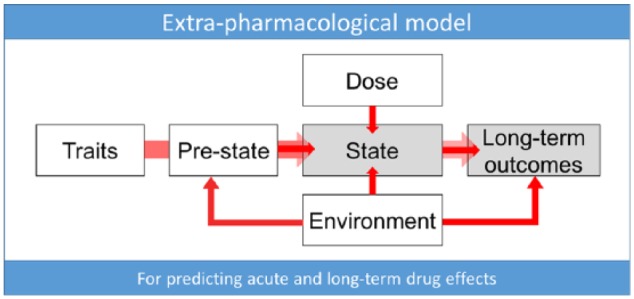
Extra-pharmacological (EP) model of drug action. This model is intended to provide a comprehensive account of the action of psychoactive drugs that takes into account important extra-pharmacological components such as trait, pre-state, dosage and environmental factors and how these interact with a given drug’s specific pharmacology to predict the quality of the acute ‘intoxicated’ or ‘medicated’ state and subsequent longer-term outcomes. The model is conceived with acute dosing in mind; however, it could also be adapted and applied to chronic dosing regimens.

**Table 1** summarises findings that support various associations between 5-HT, signalling at its post-synaptic 5-HT1A and 5-HT2A receptors and relevant psychological phenomena. A number of these associations require qualification, e.g. 5-HT2AR agonism can have opposite acute and longer-term effects (Carhart-Harris et al., 2016c). To account for this, we use the acronyms ‘ST’ and ‘LT’ for acute (short-term) and long-term outcomes respectively, where we feel disambiguation is required. Also, receptor signalling may increase plasticity in one region but decrease it in the other (e.g. Vaidya et al., 1997). As this matter is most relevant in relation to molecular markers of plasticity in the hippocampus and cortex, we use the acronyms ‘hip’ and ‘cx’ to provide the necessary disambiguation. Regarding plasticity, we use ‘general plasticity’ (gP) to refer simply to an increased capability for change and ‘regional plasticity’ (rP) when we are specifically referring regional changes in molecular markers of plasticity such as trophic factors. It is important to stress that the effects of 5-HT2AR agonism are highly context sensitive (see Figure 2), e.g. the effects of 5-HT2AR signalling on mood and mental health are likely highly sensitive to the quality of the environment in which a 5-HT2AR-mediated experience occurs (Johnson et al., 2008), and this rule may also apply for treatment with an SSRI (Branchi, 2011) perhaps due to increased 5-HT2A receptor signalling through increased synaptic 5-HT. For this reason, and due to the still developing evidence base for psychedelics for depression (e.g. see Carhart-Harris and Goodwin, 2017), we took the modest step of not describing the association between 5-HT2AR signalling and depression as ‘strong’ (+++). In fact, we describe all associations between 5-HT, mood and depression as resting on ‘reasonable’ (i.e. ++) evidence because we acknowledge that these associations are especially complex. Also, some aspects of cognition but not others may be enhanced by increased signalling at a specific receptor and this is not qualified in the table. The reader may therefore notice some contradictory associations, simply because the data are not straightforward in supporting one particular direction. Importantly, this table is not intended as an exhaustive nor comprehensive account of literature pertaining to brain serotonin function but rather as an overview of significant associations between 5-HT, its 1A and 2A receptors and specific psychological phenomena of interest. This table cannot be considered a substitute for a detailed reading of the surrounding text. To properly understand the relevant associations, a careful reading of the text and supporting references is encouraged. Key: to provide a qualitative index of the perceived strength of evidence for a given association, we use the symbols +, ++ and +++ to denote ‘weak’, ‘reasonable’ and ‘strong’ evidence. Moreover, strong associations are shown in bold font. The ‘↑’ symbol denotes an increase in a particular factor and ‘↓’ denotes a decrease. The ‘→’ symbol denotes that one factor causes another.

**Table 1.** Functions associated with brain serotonin.

	5-HT implicated	Post-synaptic (pst) 5-HT1AR signalling (sg) implicated	5-HT2AR signalling (sg) implicated
**Impulsivity and aggression (I&A)**	**5-HT ↓ → I&A ↑ +++**	**pst5-HT1ARsg↑ → I&AG↓ +++**	5-HT2ARsg↑ → I&A ↑(ST) ++ 5-HT2ARsg↑ → I&A ↓ (LT) ++
**Anxiety and stress (A&S) and punishment (Pun)**	**5-HT ↓ → A&S ↑ +++** **Pun ↑ → 5-HT ↑ → +++**	**pst5-HT1ARsg↑ → A&S ↓ +++**	5-HT2ARsg↑ → A&S ↑(ST) ++ 5-HT2ARsg↑ → A&S ↓(LT) ++
**Learning and cognition (L&C)**	5-HT ↓ → L&C ↓ ++	pst5-HT1ARsg↑ → L&C ↓ ++ pst5-HT1ARsg↑ → L&C ↑ +	5-HT2ARsg↑ → L&C ↑(ST) + 5-HT2ARsg↑ → L&C ↓(ST) ++ **5-HT2ARsg↑ → L&C ↑(LT) +++**
Depression (D) and mood*	5-HT ↓ → mood ↓ ++ 5-HT ↑ → mood ↑ ++	pst5-HT1ARsg↑ → D ↓ ++	5-HT2ARsg↑ → D ↓(LT) ++
**General plasticity (gP) and regional specific plasticity (rP)**	**5-HT ↑ → gP ↑ +++**	pst5-HT1ARsg↑ → GP ↑(hip) ++	5-HT2ARsg↑ → rP ↑(LT, cx) ++ 5-HT2ARsg↑ → rP ↓(LT, hip) ++ **5-HT2ARsg↑ → gP ↑(ST & LT) +++**

#### 5-HT2AR signalling, impulsivity and aggression

In contrast to what is typically associated with postsynaptic 5-HT1AR agonism, there is some evidence in rodents that 5-HT2AR agonism increases impulsivity (Anastasio et al., 2015; Carli et al., 2006; Winstanley et al., 2004). However, the relationship between the 5-HT2AR and impulsivity and aggression in humans is somewhat ambiguous (da Cunha-Bang et al., 2013; van Wel et al., 2012) and anti-impulsivity effects of 5-HT2AR antagonists may be an epiphenomenon of these compounds’ mild sleep-promoting/sedating properties (Ivgy-May et al., 2015; Morairty et al., 2008). Moreover, 5-HT2AR agonist psychedelics such as LSD and psilocybin are not typically associated with aggressive or impulsive behaviours in humans, and may even possess some pro-social properties in certain contexts (Dolder et al., 2016; Kraehenmann et al., 2016; Preller et al., 2016) – see also (Watts et al., 2017). Rare cases of behavioural disinhibition and even aggression have been observed with high doses of potent psychedelic 5-HT2AR agonists – but such incidences are likely to be strongly context specific (Gee et al., 2016). See Figure 2.

### Anxiety and stress

#### 5-HT1AR signalling, anxiety and stress

Related to the hypothesis that 5-HT functions to moderate aversive mental states (Deakin and Graeff, 1991) and promote patience (McDannald, 2015) is the notion that 5-HT plays an important role in negatively modulating anxiety (Piszczek et al., 2015). Selective reductions of 5-HT in the forebrain have been found to enhance anxiety-related behaviours (Pum et al., 2009; Tu et al., 2014), whereas chronically administered SSRIs have been found to reduce anxiety (Blanco et al., 2013). Like impulsivity and aggression, anxiety appears to be negatively modulated by 5-HT1AR stimulation (Heisler et al., 1998; Parks et al., 1998; Schreiber and De Vry, 1993; Toth, 2003), and although there are some contradictory findings (File et al., 1996), this effect appears to be mediated by postsynaptic 5-HT1AR signalling (Celada et al., 2013a; Gross et al., 2002; Piszczek et al., 2015; Stefanski et al., 1993; Tauscher et al., 2001; Tu et al., 2014; Zhou et al., 2008, 2014).

Postsynaptic 5-HT1A receptors are densely expressed in limbic regions and particularly the hippocampus (Pazos and Palacios, 1985; Varnas et al., 2004), which is known to be involved in anxiety (Gray, 1983; Tu et al., 2014). Serotonin 1A receptors are highly expressed on excitatory neurons in the hippocampus (Pompeiano et al., 1992) and 5-HT1AR stimulation has an inhibitory influence on pyramidal neuron activity (Andrade, 2011). Hippocampal hyperactivity is strongly associated with states of anxiety and stress (Engel et al., 2009) and 5-HT appears to quell limbic hyperactivity via the inhibitory action of postsynaptic 5-HT1ARs (Dong et al., 1998; Tada et al., 2004). This mechanism could explain the reduced metabolism and blood flow observed in limbic regions with acutely administered MDMA (Carhart-Harris et al., 2015b; Gamma et al., 2000), buspirone (Friston et al., 1991), fenfluramine (thalamus and temporal cortex (Meyer et al., 1996)) and chronically administered SSRIs (Mayberg et al., 2000) – as well as reduced cortico-limbic reactivity to negative stimuli with MDMA (Bedi et al., 2009; Carhart-Harris et al., 2014d) and SSRIs (Arnone et al., 2012; Ma, 2015). The improved ability to tolerate negative stimuli with both acute MDMA (Carhart-Harris et al., 2014d; Mithoefer et al., 2011, 2013) and chronic SSRI treatment (Corchs et al., 2009; Mineur et al., 2015) may be due to elevated levels of synaptic 5-HT activating inhibitory postsynaptic 1A receptors in stress-sensitive limbic regions. It is also likely to explain the use of SSRIs and direct 5-HT1AR agonists such as buspirone, as anxiolytic medications. There is also compelling evidence through 5-HT1AR knock out studies that this receptor is involved in the moderation of anxiety (Chattopadhyay, 2007).

#### Punishment, 5-HT release and 5-HT1AR signalling

Intriguingly, other than pharmacological manipulations (Bradbury et al., 2013), *punishment* is one of the most effective means of stimulating 5-HT release (Adell et al., 1997; Amat et al., 1998; Bland et al., 2003a, 2003b; Ferres-Coy et al., 2013; Gronli et al., 2007; Kawahara et al., 1993; Rex et al., 2005; Yoshioka et al., 1995). Several studies have demonstrated that anxiety (Rex et al., 2005) and stress (Fujino et al., 2002) can profoundly increase synaptic 5-HT. Consistent with previous theories (Deakin, 2013), it seems reasonable to suppose that brain 5-HT functions to alleviate psychological distress under adverse conditions – thereby improving coping and resilience. The moderation of aversive mental states may be evolutionarily advantageous in certain contexts, e.g. promoting a more patient, waiting and observing behavioural style, and perhaps greater sociability (or at least reduced anti-sociability). We suggest that this function is mediated by postsynaptic 5-HT1AR signalling, serving to quell hyperactivity in stress-sensitive circuits (Puig and Gulledge, 2011), particularly under conditions of mild-moderate adversity. We link this to the notion of ‘passive coping’, since the behavioural outcome is one of improved endurance of adversity via a moderation of stress and perhaps emotional responsiveness more generally (McCabe et al., 2010; Price et al., 2009).

#### Anxiety, stress and the 5-HT2AR

The serotonin 2A receptor has also been implicated in anxiety. Serotonin 2A receptor knock-out mice display reduced anxiety which is normalised when its functioning is recovered (Weisstaub et al., 2006). These findings suggest that 5-HT2AR signalling has an anxiogenic effect that is opposite to the anxiolytic effect of postsynaptic 5-HT1AR activation. This idea is leant support by findings of reduced anxiety with 5-HT2AR antagonism (Bressa et al., 1987). Serotonin 2A receptor agonists have complex effects on anxiety in humans (Zanoveli et al., 2005). Subjective anxiety is inconsistently and only marginally increased by the 5-HT2AR agonists psilocybin and LSD during their acute intoxication state (Carhart-Harris et al., 2012a, 2015a; Griffiths et al., 2006) (although acute panic can occur (Barrett et al., 2016; Carbonaro et al., 2016)), yet there is increasing evidence that anxiety can be significantly reduced for a prolonged period after a therapeutically mediated psychedelic drug experience (Gasser et al., 2014, 2015; Griffiths et al., 2016; Grob et al., 2011) – for a discussion of this apparent paradox see (Carhart-Harris et al., 2016c). Thus, whereas postsynaptic 5-HT1AR activation appears to moderate anxiety and stress, the effect of 5-HT2AR activation is more complex (Carhart-Harris et al., 2016c). Similarly, 5-HT2C receptor agonism has been associated with anxiety (and inversely with ‘assertiveness’ in rats) – but a more detailed discussion of 5-HT2C receptor functioning is beyond the remit of this paper (see Mitchell, 2005 for a relevant review).

#### The effects of 5-HT2AR signalling are highly context sensitive

In forthcoming sections, we develop the idea that 5-HT2AR signalling has a time and context sensitive effect on cognition and emotion, increasing plasticity-related processes (and often anxiety (Griffiths et al., 2006)) in the short-term while facilitating openness, learning and well-being in the longer-term (Carhart-Harris et al., 2016c; MacLean et al., 2011). If mediated properly (e.g. with appropriate psychological support and positive environmental conditions) the acute labile state can be used to facilitate emotional approach and eventual acceptance with potentially enduring beneficial effects (Roseman et al., 2017b; Watts et al., 2017); moreover, it remains possible that reduced anxiety and improved general well-being during the post-acute ‘after glow’ (Winkelman et al., 2014) of a psychedelic experience is related to agonist-induced 5-HT2AR downregulation (Buckholtz et al., 1990).

Consistent with a recent hypothesis on the function of brain 5-HT (Branchi, 2011), we predict that the plasticity-enhancing effects of 5-HT accentuate the influence of environmental factors on the individual (Branchi, 2011) but we would qualify this relationship by emphasising that it is primarily a 5-HT2AR-mediated process. Thus, we propose that 5-HT2AR signalling opens a window of plasticity during which environmental-sensitivity is enhanced and significant therapeutic work can be done. Supporting this hypothesis, central 5-HT2ARs expression is highest during key developmental periods (Sheline et al., 2002; Volgin et al., 2003) when plasticity-related learning is maximal. The quality of a 5-HT2AR dependent psychedelic experience is known to be highly sensitive to the context in which it occurs (Hartogsohn, 2016) and to be consequently predictive of long-term mental health outcomes (Carhart-Harris et al., 2017; Roseman et al., 2017a).

#### Extra-pharmacological model of drug effects

The extra-pharmacological or ‘EP’ model presented in Figure 2 is inspired by recent empirical and theoretical work on the psychedelic state and is conceived as a working model for testing and refining our understanding of the many determinants of the acute and longer-term effects of psychoactive drugs in general, albeit with special reference and relevance to psychedelics. *Trait* factors may be biological (e.g. receptor polymorphisms (Ott et al., 2006)) or psychological in nature (e.g. personality (MacLean et al., 2011) or suggestibility (Carhart-Harris et al., 2015a)). The *pre-state* refers to such thing as anticipatory anxiety, expectations and assumptions (which account for so-called ‘placebo’ and ‘nocebo’ effects), and readiness to surrender resistances and ‘let go’ to the drug effects (e.g. see Russ and Elliott, 2017). In the context of psychedelic research, the *pre-state* is traditionally referred to as the ‘set’ (Hartogsohn, 2016). *State* refers to the acute subjective and biological quality of the drug experience and may be measured via subjective rating scales or brain imaging (see Roseman et al., 2017). *Dose* relates to the drug dosage – which may be a critical determinant of *state* (Griffiths et al., 2011; Nour et al., 2016) – as well as long-term outcomes (Roseman et al., 2017). *Environment* relates to the various environmental influences. In the context of psychedelic research this is traditionally referred to as ‘setting’ (Hartogsohn, 2016). We recognise that the environment can be influential at all stages of the process of change associated with drug action. The *long-term outcomes* may include such things as symptoms of a specific psychiatric condition such as depression – measured using a standard rating scale (Carhart-Harris et al., 2016a) as well as relatively pathology-independent factors such as personality (MacLean et al., 2011) and outlook (Nour et al., 2017). The EP model may prove useful in future studies of psychedelics that aim to determine the weighting or relative influence of different predictor variables on the quality of the acute state and longer-term outcomes. Predictor variables such as *trait, pre-state, dose* and *environment* could be entered as independent variables in a regression model, with *state* as the dependent variable. Similarly, a regression model could include *state* as an independent ‘predictor’ variable, with a *long-term outcome* as the dependent variable (for example as in Roseman et al., 2017a; Russ and Elliot, 2017). This model could eventually be used to assist screening for psychedelic therapy and inform on how the therapy is to be delivered, e.g. what dose to administer and how to tune the environment to promote optimal outcomes.

### Learning and cognition

#### 5-HT1AR signalling learning and cognition

Postsynaptic 5-HT1AR stimulation is generally considered to be a desirable property of anxiolytic and antidepressant medications (Artigas, 2015), and the postsynaptic 5-HT1AR is thought to be the principal (therapeutic) site of action of SSRIs (Artigas, 2013a, 2015; Samuels et al., 2015). Chronic treatment with SSRIs has been associated with increased neurogenesis (Boldrini et al., 2009), particularly in the hippocampus (Boldrini et al., 2009, 2012) and some improvements in learning and cognition (Bui et al., 2013), albeit with some contradictory findings (Deakin et al., 2004). There is evidence to suggest that increased neurogenesis (at least in the hippocampus) is a 5-HT1AR-mediated effect (Gould, 1999; Huang and Herbert, 2005; Malberg et al., 2000; Santarelli et al., 2003); however, other 5-HT receptors (e.g. the 5-HT4 and 5-HT2A) are also thought to contribute (Azmitia, 2001; Imoto et al., 2015; Jha et al., 2008; Kraus et al., 2017).

Despite this association between 5-HT1AR signalling and neurogenesis, there is a body of evidence to suggest that postsynaptic 5-HT1AR stimulation is impairing to learning and cognition (Ogren et al., 2008), so how can we reconcile these things? One possibility is that the observed pro-cognitive effects of SSRIs are actually mediated by other (non-1A) 5-HT receptors (Boulougouris et al., 2008; Furr et al., 2012; Imoto et al., 2015), and another is that improvements in cognition in patients treated with SSRIs is an epiphenomenon of improvements in mood (Chepenik et al., 2007). It is also important to note that the evidence that SSRIs improve cognition is relatively weak (Beheydt et al., 2015; Knorr, 2012; Knorr et al., 2011; Siepmann et al., 2003) and their modest ability to address cognitive symptoms in depression is considered one of their limitations (Popovic et al., 2015).

#### 5-HT2AR signalling, learning and cognition

The relationship between the 5-HT2AR and cognition is somewhat different to that of the 5-HT1AR. As discussed above, activation of postsynaptic 5-HT1ARs is associated with cognitive and learning impairments (Ogren et al., 2008), whereas 5-HT2AR activation is associated with improvements in certain aspects of cognition and learning (Gimpl et al., 1979; Harvey, 1996, 2003; Harvey et al., 2004, 2012; King et al., 1974; Romano et al., 2006, 2010; Welsh et al., 1998; Zhang and Stackman, 2015; Zhang et al., 2016) as well as an unlearning or ‘extinction’ learning (Zhang et al., 2013). Serotonin 2A receptor activation has also been associated with neurogenesis (Catlow et al., 2013; Cavus and Duman, 2003; Frankel and Cunningham, 2002; Gewirtz et al., 2002; Jones et al., 2009; Meller et al., 2002; Niitsu et al., 1995; Vaidya et al., 1997), particularly in the cortex (Gewirtz et al., 2002; Jones et al., 2009; Vaidya et al., 1997) (but not in the hippocampus (Vaidya et al., 1997)), which may explain the type of cognitive and learning enhancements that are associated with its functioning (e.g. associative learning). Specifically, a number of studies have shown enhancements of associative learning with 5-HT2AR agonism and impairments with its blockade (Barre et al., 2016; Harvey, 1996, 2003; Harvey et al., 2004; Romano et al., 2000, 2006; Welsh et al., 1998).

Cognitive flexibility in humans is thought to be positively modulated by 5-HT2AR functioning (Boulougouris et al., 2008) and there is evidence to suggest that 5-HT2AR agonists (such as LSD and psilocybin) enhance cognitive flexibility and creative thinking (Frecska et al., 2012; Harman et al., 1966; Janiger and Dobkin de Rios, 1989; King et al., 1974; MacLean et al., 2011; McGlothlin et al., 1967; Sessa, 2008), potentially in an enduring way (MacLean et al., 2011). Serotonin depletion and inactivation has been shown to impair cognitive flexibility (Clarke et al., 2004, 2007; Matias et al., 2017) and there is evidence that this may be due to decreased basal activation of 5-HT2ARs (Boulougouris et al., 2008; Furr et al., 2012). Serotonin neurons have been found to activate when animals experience a surprising violation of assumptions, independent of its reward-related implications (Matias et al., 2017), supporting the association between 5-HT, environmental sensitivity and adaptability (Branchi, 2011). Our argument here is that 5-HT2AR signalling is the key mediator of this effect. Promotion of plasticity via 5-HT2AR signalling is central to our thesis that, along with improving stress-tolerance, a key function of brain serotonin transmission is to engage processes necessary for change, when change is necessary. Note: although we acknowledge it would be pertinent and potentially valuable, a more in-depth discussion of the 5-HT2AR and animal and human behavioural measures of cognitive flexibility is beyond the scope of this paper.

### Serotonin, depression and mood

#### Evidence for an association between serotonin and mood

Serotonin was first isolated and named in the late 1940s (Rapport et al., 1948) and subsequently found in the brain in the early 1950s (Gaddum, 1953; Twarog and Page, 1953). At the same time, scientists were beginning to identify interactions between serotonin and the recently discovered lysergic acid diethylamide (LSD) (Gaddum, 1953; Shaw and Woolley, 1956). Struck by LSD’s remarkable potency (psychoactive in doses as low as 20 µg) and powerful modulatory effects on mood and cognition (Busch and Johnson, 1950; Hofmann, 1980), it was speculated that abnormal serotoninergic functioning may underlie certain mental disorders (Gaddum, 1957; Woolley and Shaw, 1954). Although the ‘psychotomimetic’ (mimicking psychosis) properties of LSD and related psychedelics were recognised in the 1950s and 60s (Isbell et al., 1959), as they are today (Carhart-Harris et al., 2013a, 2016c), these compounds were also used extensively as psychotherapeutic aids for the treatment of a range of disorders, including depression and anxiety (Grinspoon and Bakalar, 1979; Sandison, 1954; Sandison and Hopkin, 1964).

The earliest and most direct evidence for the involvement of monoamines in mood regulation however, came with the observation that reserpine, which depletes 5-HT and noradrenaline in the brain (Pletscher et al., 1955), also induces depressed mood in some individuals (Achor et al., 1955) – see also (Antkiewicz-Michaluk et al., 2014). This observation was closely followed by the discovery of the antidepressant properties of the monoamine oxidase inhibitors (MAOIs) (Udenfriend et al., 1957) and subsequently the tricyclic antidepressants (TCAs) (Axelrod and Inscoe, 1963; Kuhn, 1958) – both of which increase synaptic monoamines (Gur et al., 1999; Matos et al., 1990). More specific evidence for the involvement of 5-HT in depression came from studies showing a combined antidepressant effect with an MAOI plus tryptophan, the biochemical precursor to 5-HT (Coppen et al., 1963; Hess and Doepfner, 1961; Pare, 1965).

The idea that serotonergic mechanisms are involved in the pathogenesis and treatment of depression was controversial in the 1960s (Coppen, 1969, 1967); however, it gradually gained traction in the 1980s and into the 1990s with the development and licensing of the SSRIs (Carlsson, 1981; Cowen and Browning, 2015) and particularly fluoxetine (Bremner, 1984). When chronically administered, SSRIs increase concentrations of synaptic 5-HT (Smith et al., 2000) by blocking its reuptake (Carlsson, 1981), show superior efficacy to placebo in depression (Horder et al., 2011; Hieronymus et al., 2016; Barth et al., 2016) and are safer than MAOIs and TCAs (Pletscher, 1991). Another important finding supporting the involvement of serotonin in depression was the observation that acute tryptophan depletion can induce a (transient) relapse in symptoms in formerly depressed patients (Smith et al., 1997) and plasma tryptophan levels have been found to be low in patients with severe depression (Anderson et al., 1990), potentially owing to inflammation-related mechanisms (Wichers et al., 2005).

The involvement of serotonin in mood regulation is further substantiated by the fact that the potent mood-enhancing agent, MDMA, has marked 5-HT releasing properties (Bradbury et al., 2013). In rodents, MDMA is also a noradrenaline (NA) and dopamine (DA) releaser (Kankaanpaa et al., 1998) but its 5-HT releasing properties are far more pronounced (Bradbury et al., 2013; Golembiowska et al., 2016). Blockade of the serotonin transporter by pre-treatment with the SSRI citalopram, significantly attenuated the signature positive mood effects of MDMA (Liechti and Vollenweider, 2000, 2001) – presumably via preventing MDMA from interacting with the 5-HT transporter. Pre-treatment with the D2 antagonist haloperidol also attenuated the positive mood effects of MDMA (Liechti and Vollenweider, 2001) – suggesting that combined DA and 5-HT functioning may have a synergistic influence on mood. However, in a separate study, combining the DA reuptake blocker methylphenidate with MDMA did not have a supplementary influence on positive mood (Hysek et al., 2014b) and stimulants with greater DA than 5-HT releasing properties (such as amphetamine, cocaine and methylphenidate) do not induce the same pro-empathy and pro-social sentiments as well as frank euphoria that can be attributed to MDMA (Bedi et al., 2014; Schmid et al., 2014). The sudden popularity of mephedrone as a party-drug in the early 2010s (Carhart-Harris et al., 2011), may be explained by its pronounced serotonin-releasing properties (Golembiowska et al., 2016), in conjunction with DA release (Kehr et al., 2011), with users likening its euphoric effect to that of MDMA (Carhart-Harris et al., 2011). Like MDMA, mephedrone causes massive 5-HT release that far exceeds its still considerable DA releasing properties (Golembiowska et al., 2016).

In summary, there is a wealth of evidence that 5-HT is involved in the regulation of mood but exactly how it does this is not properly understood (Dayan and Huys, 2015). A central theme of this paper is that the combination of 5-HT1A and 5-HT2A receptor signalling has a complementary effect on mood by promoting stress moderation and patience (predominantly 5-HT1AR mediated) and plasticity and open-mindedness (predominantly 5-HT2AR mediated). For the remainder of the paper, these ideas will be unpacked, first with a focus on postsynaptic 5-HT1AR signalling, before addressing the function of 5-HT2AR signalling in detail.

#### Postsynaptic 5-HT1AR signalling and mood

The importance of postsynaptic 5-HT1AR receptor signalling in the therapeutic action of serotonergic antidepressants has been convincingly demonstrated (Blier and Ward, 2003; Blier et al., 1997). Selective 5-HT1AR agonists appear to work in a similar way to traditional serotonergic antidepressants (Lucki, 1991), i.e. with a delayed onset of action of 7–14 days due to the gradual desensitisation of the presynaptic 5-HT1A autoreceptors (Blier and Ward, 2003). Subsequent to autoreceptor desensitisation (Le Poul et al., 1995), 5-HT1AR agonists (such as buspirone) appear to act in the same stress-reducing way as has been described for the SSRIs, and this may explain their therapeutic value as anxiolytics (Beneytez et al., 1998; Celada et al., 2013a; Chilmonczyk et al., 2015; Gordon and Hen, 2004; Jolas et al., 1995; Koek et al., 1998; Li et al., 2006; Plaznik et al., 1994; Strauss et al., 2013). Moreover, 5-HT1AR knock-out rodents exhibit greater levels of anxiety and depressive symptoms (Heisler et al., 1998; Ramboz et al., 1998), presumably due to deficient postsynaptic 5-HT1AR-signalling (e.g. in limbic regions).

Determining the importance of the 5-HT1AR to the mechanisms of action of MDMA and classic psychedelics is difficult, due to the unavailability of selective 5-HT1AR antagonists for human research which could be given as blocking agents. The non-selective weak 5-HT1AR antagonist pindolol had a negligible influence on MDMA’s positive mood effects in one study (van Wel et al., 2012) but slightly attenuated them in another (Hasler et al., 2009). Pindolol slightly augmented the psychoactive effects of the classic psychedelic and 5-HT2AR agonist dimethyltryptamine (DMT) (Strassman, 1996), and the 5-HT1AR partial agonist buspirone significantly attenuated the psychoactive effects of psilocybin (Pokorny et al., 2016). The lack of pharmacological selectivity and/or only partial agonism and weak antagonism of buspirone and pindolol (respectively) preclude us from making strong inferences about their effects in pre-treatment studies, although broadly speaking, they support a view that postsynaptic 1A receptor signalling is only mildly (Hasler et al., 2009) and unreliably (van Wel et al., 2012) involved in MDMA’s positive mood effects but may significantly attenuate some of the key psychological effects of classic psychedelics (Pokorny et al., 2016; Strassman, 1996). Supporting this latter inference, depletion of brain serotonin augments the behavioural effects of LSD in animals (Harvey et al., 1975) and humans (Resnick et al., 1965) and this effect may be explained in part by lower postsynaptic 5-HT1AR signalling enabling an exaggerated effect at the 5-HT2A receptor, although an adaptive, homeostatic upregulation of 5-HT2AR availability due to low synaptic 5-HT may be another mechanism (Jennings et al., 2008, 2016). Note also that 5-HT1AR expression is low in the visual cortex (Figure 1) which may explain why 5-HT2AR agonist psychedelics have pronounced visual perceptual effects – i.e. because the excitatory effects of 5-HT2AR agonism go unopposed (by 5-HT1AR signalling) in this region.

Further considering the contribution of 5-HT1AR signalling to MDMA’s acute effects, it is notable that marked changes in cerebral blood flow and functional connectivity in limbic structures (that exhibit the richest expression of 5-HT1A receptors in the forebrain) were observed with acute MDMA administration (Carhart-Harris et al., 2015b), and MDMA’s characteristic pro-social effects were significantly attenuated by pre-treatment with a selective 5-HT1AR antagonist in rats (Hunt et al., 2011) (although see Pitts et al., 2017). The development of new PET ligands sensitive to 5-HT release may prove useful in determining the contribution of different receptor subtypes to the psychological effects of MDMA and other potent serotonergic drugs (Jorgensen et al., 2016; Tyacke and Nutt, 2015). However, in brief, it is our assumption that the effects of MDMA reflect combined signalling at postsynaptic 5-HT1AR, 5-HT2AR and catecholamine receptors (i.e. DA and NA) to produce a state of improved stress tolerability (5-HT1AR-mediated) combined with increased cognitive flexibility and emotional lability (5-HT2AR-mediated) and enhanced focus, motivation and confidence (NA/DA receptor mediated) that in combination, is especially conducive to positive mood (Sessa, 2016).

#### 5-HT2AR signalling, depression and mood

It has been convention in neuropsychopharmacology to view 5-HT2AR agonism as potentially harmful (or at least unconducive) to mental health. The main arguments for this are: (1) 5-HT2AR agonists, such as LSD and psilocybin, are psychotomimetics (i.e. psychosis models) (Curran et al., 2009; Gerber and Tonegawa, 2004); and (2) a number of antidepressants (Carpenter et al., 1999) as well as many antipsychotics (Meltzer, 2012) have 5-HT2AR antagonist properties. However, recent studies have begun to challenge the notion that 5-HT2AR agonism is an undesirable property for a psychotropic medication (Carhart-Harris et al., 2016c; Griffiths and Grob, 2010; Carhart-Harris et al., 2016b; Qesseveur et al., 2016; Petit et al., 2014 – see Carhart-Harris and Goodwin, 2017 for a review) – and about their harm, comparative rating scales suggest 5-HT2AR agonist psychedelics like psilocybin are among the least harmful drugs of potential misuse (Carhart-Harris and Nutt, 2013; Nutt et al., 2010; van Amsterdam et al., 2015). Moreover, an increasing number of studies are reporting enduring positive mental health outcomes (Bogenschutz et al., 2015; Bouso et al., 2012; Gasser et al., 2014; Grob et al., 2011; Hendricks et al., 2015b; Osorio Fde et al., 2015) and psychological well-being (Carhart-Harris et al., 2016c; Griffiths et al., 2008) with administration and use of 5-HT2AR agonist psychedelics. Additionally, several studies have found associations between 5-HT2AR polymorphisms and SSRI response (Kishi et al., 2010; McMahon et al., 2006; Wilkie et al., 2009), although it is unclear if alleles predicting better response are associated with more or less 5-HT2AR functioning. Potentially, resolving this, however, a recent study suggested that 5-HT2AR signalling is an important (and therefore underappreciated) component of antidepressant action of SSRIs (Qesseveur et al., 2016).

Supporting the principle that 5-HT2AR agonism is a viable antidepressant target, are the growing number of studies demonstrating the antidepressant potential of 5-HT2AR agonist psychedelics (Baumeister et al., 2014; Buchborn et al., 2014; Carhart-Harris et al., 2016b; Griffiths et al., 2016; Grob et al., 2011; Osorio Fde et al., 2015; Ross et al., 2016; Sanches et al., 2016 – see Carjart-Harris and Goodwin, 2017 for a review). For example, a recent pilot study by our team reported rapid and enduring improvements in depressive symptoms after two treatment sessions with psilocybin in patients with treatment-resistant depression (Carhart-Harris et al., 2016b). The results of this study are consistent with those of others reporting reduced depressive symptoms in depressed patients treated with ayahuasca (Osorio Fde et al., 2015; Sanches et al., 2016) and end-of-life anxiety patients treated with psilocybin (Griffiths et al., 2016; Grob et al., 2011; Ross et al., 2016), as well as a population study showing lower rates of psychological distress and suicidality in relation to psychedelic drug use (Hendricks et al., 2015b). Taken together, these findings motivate a revision of the conventional view that psychedelics are harmful to mental health (Hendricks et al., 2015b), and encourage a rethink on the role of 5-HT2AR signalling in the pharmacology of depression (see also (Petit et al., 2014; Qesseveur et al., 2016).

Further support for a positive association between 5-HT2AR signalling and (trait) psychological health comes from human PET imaging work that has shown a positive relationship between 5-HT2AR binding and trait neuroticism (Frokjaer et al., 2008), pessimism (Bhagwagar et al., 2006; Meyer et al., 2003) and personality disorder (Soloff et al., 2007; Rosell et al., 2010). Cortical 5-HT2AR expression is sensitive to basal 5-HT concentrations (Cahir et al., 2007; Jorgensen et al., 2016), with 5-HT2A receptors becoming more populous and/or available in response to reduced synaptic 5-HT (Cahir et al., 2007; Jennings et al., 2008; Jorgensen et al., 2016) and less available in response to increased synaptic 5-HT (Jorgensen et al., 2016; Meyer et al., 2001). Thus, increased 5-HT2AR binding and associated pessimistic thinking (Bhagwagar et al., 2006; Meyer et al., 2003) may be a corollary of deficient 5-HT2AR signalling – and the enduring increases in optimism that have been observed with LSD (Carhart-Harris et al., 2016c) may be viewed as evidence of extreme 5-HT2AR signalling having a lasting impact on positive thinking (Carhart-Harris et al., 2016c).

Postmortem studies showing increased 5-HT2AR availability in unmedicated depressed patients (Shelton et al., 2009) and suicide victims (Anisman et al., 2008; Pandey et al., 2002; Stanley and Mann, 1983; Turecki et al., 1999) could be viewed as consistent with the hypothesis that there is an adaptive upregulation of 5-HT2A receptors in response to deficient 5-HT2AR signalling in depression. The existent of discrepant findings (e.g. decreased 5-HT2AR availability in depression and suicide victims) that challenge this hypothesis may be explained by the confounding influence of antidepressant and other psychiatric medications – which reverse this relationship by downregulating 5-HT2AR availability (Attar-Levy et al., 1999; Dean et al., 2014; Gray and Roth, 2001; Muguruza et al., 2014; van Heeringen et al., 2003; Yatham et al., 1999).

#### Electroconvulsive shock and 5-HT2AR functioning

The effect of electroconvulsive shock (ECS) on 5-HT2AR densities and functioning is important to address, particularly given the notable efficacy of electroconvulsive therapy (ECT) in terms of reducing depressive symptoms for a period (UK ECT Review Group, 2003). Interestingly, we have recently found that functional brain changes one day after psilocybin for treatment-resistant depression compare best with those of ECT (Carhart-Harris et al., 2017b). For example, as with ECT (Bolwig, 2015), the post-psilocybin treatment brain changes were the inverse of what is typically seen during the acute psilocybin experience itself (Carhart-Harris et al., 2017b). More specifically, whereas resting state functional connectivity in the default-mode network is significantly decreased during the acute psychedelic experience (Carhart-Harris et al., 2016), it is increased (or ‘normalised’) one day after psilocybin for treatment-resistant depression – and this effect is greatest in treatment responders (Carhart-Harris et al., 2017b). Increased or ‘normalised’ DMN RSFC has also been seen after successful treatment with ECT (Mulders et al., 2016).

Early rat work revealed increased 5-HT2AR functioning (Moorman et al., 1996) and cortical 5-HT2AR expression after ECS (Burnet et al., 1995, 1999; Butler et al., 1993) – an effect that appeared to be relatively selective for the 5-HT2AR in relation to other serotonin receptor subtypes (Burnet et al., 1999). However, contradictory findings have since been observed in primates (Strome et al., 2005) and humans (Yatham et al., 2010) with 5-HT2AR binding showing decreased post ECS/ECT. This downregulation of 5-HT2AR densities post ECT is more consistent with the effects of conventional antidepressant medications (Yatham et al., 1999) – as well as classic psychedelics (Buckholtz et al., 1990) – and also makes more logical sense given the marked 5-HT release that is associated with ECS (Zis et al., 1992).

How do we explain the observed 5-HT2AR upregulation in rats however? Stress has been found to increase 5-HT2AR density (Katagiri et al., 2001) and affinity (Harvey et al., 2003) in rats. Extreme stress is hypothesised to engage ‘pathway 2’ in our bipartite model, which is mediated by 5-HT2AR signalling, and characterised by a rapid plasticity – serving to facilitate major change in conditions of extreme adversity. Although speculative, one interpretation of the upregulated 5-HT2AR functioning post ECS in rats, is that it is a consequence of the extreme stress (‘shock’) of the procedure in this species. It might also be worth noting that ECT has been found to promote neural plasticity (Bouckaert et al. 2014; Joshi et al. 2016), and so is consistent with pathway 2 in this regard.

#### 5-HT2A agonists and antagonists as antidepressants: resolving a paradox

**Figure 3.**
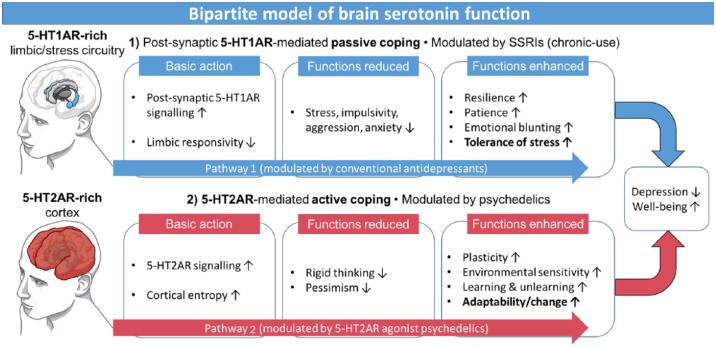
**A two-part or ‘bipartite’ model of brain serotonin function**. Model proposes that brain serotonin mediates adaptive responses to adversity via two distinct mechanisms: one mediated by postsynaptic 5-HT1AR signalling in aid of stress moderation (pathway 1) and the other mediated by 5-HT2AR signalling is aid of more substantial adaptive changes (pathway 2). SSRIs and other conventional antidepressant medications work on and can enhance pathway 1, whereas pathway 2 can be enhanced by 5-HT2AR agonist psychedelic drugs such as psilocybin. Note: it is hypothesised that active coping can be most effectively implemented if the window of plasticity afforded by 5-HT2AR agonism is complemented by supportive psychotherapy that promotes a willingness to confront and work through sources of stress (Watts et al., 2017). Illustrations by Samantha Strong (S.L.Strong1@bradford.ac.uk).

Some effective drugs for depression (such as mirtazapine) have 5-HT2AR antagonist properties (Watanabe et al., 2008) and 5-HT2AR antagonist antipsychotic drugs (such as risperidone and olanzapine) have been found to augment the antidepressant efficacy of SSRIs in treatment-resistant depression (Marangell et al., 2002; Ostroff and Nelson, 1999; Shelton and Papakostas, 2008). This has led some to consider 5-HT2AR antagonism a treatment target in depression (Pandey et al., 2010) but this matter requires some careful thought, not least because 5-HT2AR antagonism presents additional side-effects to those of first-line antidepressants such as SSRIs (Jarema, 2007; Shelton and Papakostas, 2008; Teegarden et al., 2008). To our knowledge, selective 5-HT2AR antagonists have not been trialled as stand-alone treatments for depression, and have largely failed as stand-alone treatments for schizophrenia (Ebdrup et al., 2011), so their efficacy appears to be predicated on the augmentation of other pharmacological mechanisms. For example, blocking postsynaptic 5-HT2ARs in the mPFC may lessen the ability of top-down circuits to inhibit the firing of serotonergic neurons in the midbrain (potentially leading to increased 5-HT efflux) (Artigas, 2013a), and 5-HT2AR blockade more generally, may encourage a preferential effect of 5-HT on its postsynaptic 5-HT1A receptors. Considered in this way, the effects of 5-HT2AR antagonism could be perceived as supplementing the stress moderation effects of postsynaptic 5-HT1AR agonism, and so pathway 1 in our bipartite model (Figure 3). Moreover, 5-HT2AR antagonists have mild pro-sleep/sedating properties (Idzikowski et al., 1987; Teegarden et al., 2008; Vanover and Davis, 2010) that could complement the stress moderating effects of SSRIs.

A likely solution to the paradox that 5-HT2AR agonists and antagonists have antidepressant properties is that they achieve the same outcome but via different routes. Whereas 5-HT2AR antagonism supplements the emotionally moderating effects associated with postsynaptic 5-HT1AR signalling (pathway 1), 5-HT2AR agonism may work to enhance plasticity, adaptability and the capacity for change. Both mechanisms can be viewed as adaptive responses to adverse conditions, with potentially consistent outcomes, albeit achieved via different, perhaps even antithetical mechanisms.

#### Acute versus longer-term mood effects of 5-HT2AR signalling

The paradox that 5-HT2AR agonist psychedelics can be acutely psychotomimetic (Carhart-Harris et al., 2013a; Gouzoulis-Mayfrank et al., 2005) and yet have long-term beneficial effects on well-being (Griffiths et al., 2006) and mental health (Carhart-Harris et al., 2016a; Griffiths et al., 2008; Hendricks et al., 2015b) has previously been discussed (Carhart-Harris et al., 2016c). In brief, it has been proposed that the acute state produced by 5-HT2AR agonist psychedelics does not directly modulate the valence of mood, i.e. it does not directly promote either positive or negative mood (Carhart-Harris et al., 2016c). This argument could be contested on the basis that positive mood effects are often seen with acute administration of psychedelics (Schmid et al., 2015) and the positive mood effects of MDMA (van Wel et al., 2012), LSD (Preller, 2016), psilocybin (Kometer et al., 2012) and ayahuasca (Valle et al., 2016) are all attenuated by pre-treatment with a 5-HT2AR antagonist, as are the pro-social effects of MDMA (Pitts et al., 2017). However, anxiety and psychosis-like symptoms are also often seen acutely with psychedelics (Carhart-Harris et al., 2016c) and these can also be attenuated by 5-HT2AR antagonism (Vollenweider et al., 1998). Moreover, in studies that found enhanced mood with psychedelics, psychological preparation and support was generally provided, which helps channel the experience in a positive direction. Similarly, volunteers may have had positive expectations about their experience that biased their appraisal of the acute experience. These matters are relevant to our extra-pharmacological model presented above (Figure 2), as well as the enhanced environmental sensitivity model proposed for serotonin itself (Branchi, 2011) and 5-HT2AR signalling more specifically (pathway 2, Figure 3).

One proposed solution to this apparent paradox, is that the acute and longer-term effects of psychedelics are distinct, with the acute effects being marked by emotional arousal and *lability* (Carhart-Harris et al., 2016c; Kaelen et al., 2015) rather than positive mood per se, and longer-term changes are more reliably biased towards positive mood (perhaps somewhat analogous to near-death experiences (Greyson, 2008)) with improvements in psychological well-being (Griffiths et al., 2006; Hendricks et al., 2015a, 2015b), optimism (Carhart-Harris et al., 2016c) and openness (MacLean et al., 2011). The importance of emotional breakthrough after acute struggle may be highly relevant in this context (Watts et al., 2017), as may the occurrence of peak-type experiences (Roseman et al., 2017a), both topics we intend to study more closely in the future. Agonist-induced 5-HT2AR downregulation may also play a significant role (Buckholtz et al., 1990), at least during the after-glow period 1–2 weeks post exposure (Winkelman, 2014).

This is a complex problem for future studies to dissect. However, one way we may begin to inform on it, is to address the question of whether the acute and longer-term responses to psychedelics relate to each other – and indeed, there is already ample evidence that they do (Carhart-Harris et al., 2017a; Griffiths et al., 2016; Roseman et al., 2017a; Ross et al., 2016). A recent questionnaire study found that the psychological difficulty of an acute psychedelic experience was predictive of longer-term improvements in well-being (Carbonaro et al., 2016), although the same study also found that the *duration* of such difficulty was predictive of long-term decreases in well-being (Carbonaro et al., 2016). A number of studies have found that especially intense psychedelic experiences predict positive long-term outcomes – particularly if they contain phenomena consistent with so-called ‘mystical’ (Stace, 1961) or ‘peak’ (Maslow, 1970) experiences (Bogenschutz et al., 2015; Griffiths et al., 2008, 2016; Johnson et al., 2016; Ross et al., 2016). Moreover, a recent LSD neuroimaging study by our team found that acute ‘entropic’ brain changes under the drug (Carhart-Harris et al., 2014b) were predictive of long-term increases in the personality trait ‘openness’ (Lebedev et al., 2016). As highlighted in our EP model (Figure 2), it is important that we try to better understand how extra-pharmacological factors may interact with a drug’s direct pharmacological effects to determine the quality of an acute drug experience and ensuing long-term effects – and this is especially pertinent in the context of psychedelics.

## The function of brain 5-HT2AR signalling

### 5-HT2AR mediated plasticity

There is a growing body of evidence that enhanced 5-HT2AR signalling produces a plastic state (in the sense of an enhanced capacity for change), both psychologically (Boulougouris et al., 2008; Carhart-Harris et al., 2015a, 2016c; Clarke et al., 2007; Kaelen et al., 2015; Kuypers et al., 2016) and neurobiologically (Azmitia, 2001; Barre et al., 2016; Carhart-Harris et al., 2012a, 2014b, 2016c; Gewirtz et al., 2002; Lebedev et al., 2016; Tagliazucchi et al., 2016; Vaidya et al., 1997; Yoshinaga et al., 2013). We propose that this 5-HT2AR-mediated plasticity is of fundamental importance to the acute and longer-term action of 5-HT2AR agonist psychedelics, potentially explaining their idiosyncratic phenomenology and remarkable behavioural effects – including their ability to elicit long-term beneficial (Carhart-Harris et al., 2016a; Griffiths et al., 2011; Hendricks et al., 2015a), and (albeit less common) harmful changes (Lerner and Lev-Ran, 2015; Cohen, 1966; Iaria et al., 2010).

### Plasticity and the entropic brain

The proposal that psychedelics induce a plastic state is consistent with the ‘entropic brain’ hypothesis, introduced by us in 2014 (Carhart-Harris et al., 2014b). This idea emerged out of observations of consistencies between neuroimaging findings on the action of psychedelics (Carhart-Harris et al., 2014b; Muthukumaraswamy et al., 2013) and a sense that their physical (brain) effects recapitulate their psychological effects – and vice versa. Inspired by Karl Friston’s Free-Energy principle (Friston, 2010), the information theory-based measure of *entropy* was applied to the psychedelic state in an effort to capture its essential phenomenological and neurophysiological qualities. Entropy is formally both *uncertainty* and *unpredictability* (Ben-Naim, 2007) – and not coincidentally, these terms possess meaning in both a mechanistic *and* subjective sense. A growing number of analyses are now endorsing the principle that the brain exhibits increased entropy under psychedelics (Atasoy, 2017; Carhart-Harris et al., 2014b; Lebedev et al., 2016; Schartner et al., 2017; Tagliazucchi E, 2014; Viol, 2016) (see also Gallimore, 2015) and countless other human and animal studies by independent teams, despite not formally measuring entropy, report findings that are consistent with the entropic brain principle (Celada et al., 2013b; Muthukumaraswamy et al., 2013; Riba et al., 2004, 2014; Wood et al., 2012).

Entropy exists most purely as an index of uncertainty (Ben-Naim, 2007) but its origins lie in thermodynamics (Ben-Naim, 2007, 2008). Entropy is perhaps most familiar to people in the context of thermodynamics and specifically how it relates to the second law: that isolated systems tend towards disorder, or exhibit increased entropy over time (i.e. decay). The relationship between information theory-based entropy and thermodynamic entropy is a formal one, with the latter being merely an applied and contextualised version of the former (Ben-Naim, 2007, 2008).

In the context of 5-HT2AR signalling and how this may inform on the function of brain serotonin, one may think of enhancing 5-HT2AR signalling as analogous to increasing the temperature (or excitability) of the brain; indeed, the excitatory effect of 5-HT2AR signalling has long been recognised (Aghajanian and Marek, 1999; Celada et al., 2013b). Extending this analogy to the process of *annealing* (i.e. whereby a metal is heated to make it more malleable) – one may think of 5-HT2AR signalling as functioning to induce an entropic state characterised by enhanced flexibility and malleability during which work can be done that, upon cooling, may leave a lasting change (Gopnik, 2010). Viewed through the lens of the popular Bayesian brain model of brain function (Knill and Pouget, 2004), one could see this 5-HT2AR-mediated entropic state as working to ‘reset’ reinforced priors in depression – such as pessimistic beliefs and negative self-perceptions (Moutoussis et al., 2014). See Carhart-Harris et al. (2017b) for recent neurobiological support for this idea.

### 5-HT2AR induced plasticity mediates environmental sensitivity

The evolutionary value of neural and behavioural plasticity is well recognised (Belsky and Pluess, 2013; Boyce and Ellis, 2005), and in this context, the plasticity-mediating role of serotonin is becoming increasingly well appreciated (Alboni et al., 2017; Belsky et al., 2009; Branchi, 2011; Chiarotti et al., 2017). The importance of plasticity for learning has obvious functional value: in early life, when behaviour and cognition require considerable refinement but also in extreme adversity, when major behavioural change may be necessary for survival.

Serotonin is known to play a vital role in brain development (Azmitia, 2001; Kepser and Homberg, 2015; Lambe et al., 2011) and has been found to reverse processes of maturation, both at the cellular (Kobayashi et al., 2010; Maya Vetencourt et al., 2008) and brain network level (Carhart-Harris et al., 2016d; Tagliazucchi et al., 2016) in both cases likely via 5-HT2AR related mechanisms. Regarding 5-HT2AR signalling, fMRI studies have shown that LSD and psilocybin temporarily reverse processes of network integration and segregation that characterise the developing brain (Wylie et al., 2014), and this ‘brain regression’ is mirrored at the psychological level by a psychological regression that is characteristic of the psychedelic state (Carhart-Harris et al., 2016d; Roseman et al., 2014; Tagliazucchi et al., 2016). Consistently, processes of neuronal differentiation that occur during development were found to be aided by 5-HT1AR signalling but inhibited by 5-HT2AR signalling (Azmitia, 2001).

Crucially, 5-HT2AR signalling has been found to be highly influential during early development (Beique et al., 2004; Zhang, 2003) and to be maximal during key developmental periods (Lambe et al., 2011) suggesting that 5-HT2AR-mediated plasticity facilitates the intense learning that is needed during critical periods. Children have been found to demonstrate superior performance than adults in certain tasks requiring open-mindedness and the ‘de-weighting’ of prior knowledge (Lucas et al., 2014) and psychedelics are strongly associated with unconventional thinking (Harman et al., 1966; Kuypers et al., 2016), vivid imagery and imagination (Carhart-Harris et al., 2012b, 2015a; Kaelen et al., 2016) and suggestibility (Carhart-Harris et al., 2015a).

Trend decreases in openness appear to occur with maturation (Costa and McCrae, 1988) and 5-HT2AR availability is known to markedly decrease once adulthood has been reached (Sheline et al., 2002). Enduring increases in openness have been found after psilocybin (MacLean et al., 2011) and LSD (Carhart-Harris et al., 2016c) – remarkable findings given that personality is normally highly stable in adulthood. Trait absorption, which is related to openness and a susceptibility to become immersed and absorbed in one’s inner or outer world (Ott, 2006; Parsons et al., 2015; Tellegen and Atkinson, 1974), has been found to: (1) predict sensitivity to psilocybin’s acute effects (Studerus et al., 2012), and (2) be associated with a polymorphism linked to stronger 5-HT2AR binding (Ott et al., 2005).

There is likely to be an optimal level of cognitive and psychological flexibility for a given context (Carhart-Harris et al., 2014b) and high doses of psychedelics risk overshooting this through extreme 5-HT2AR signalling causing an excessive flexibility that is unconducive to accurate reality testing and conventional cognition and behaviour (Carhart-Harris et al., 2014b). Interestingly, recent anecdotal reports suggest that semi-regular use of very low doses of psychedelics (referred to colloquially as ‘micro-dosing’) may facilitate creative problem solving and improve mood (Gregoire, 2016; Waldman, 2017) – a claim that urgently requires empirical verification through controlled research. Reports of ‘over-view’ type insights, i.e. an improved ability to see the ‘bigger picture’ under psychedelics, are relatively common among user, participant and patient reports (Sessa, 2008; Harman et al., 1966), and ‘aha’ type insights have been described (Grof, 1975; Sandison and Hopkin, 1964; Watts et al., 2017). Moreover, acute insight experienced during treatment with psilocybin for treatment-resistant depression was recently found to be predictive of positive long-term clinical outcomes (Carhart-Harris et al., 2017a). If evidence for psychedelic-induced insight is substantiated by further research, this will have interesting implications for our understanding of optimal cognition (Carhart-Harris et al., 2014b) and the science of nootropics (Froestl et al., 2014).

Relatedly, more work is required to test the reliability of the recent finding that psychedelics tune the brain closer to criticality (Atasoy et al., 2017), and what the functional and therapeutic implications of this might be. Critical systems are known to be maximally sensitive to perturbation (Bak, 1997), and although speculative, this could account for the high sensitivity to the environment that is characteristic of the psychedelic state (Hartogsohn, 2016).

Much has been written about differential vulnerability to stress in medicine and psychiatry, e.g. the so-called stress-diathesis model of mental illness (Morley, 1983). However, recent revisions of this model possess considerable appeal, particularly when applied to the context of brain serotonin (Belsky and Pluess, 2009; Branchi, 2011). According to these revised models, greater sensitivity to the environment may translate into greater well-being if conditions be favourable, or vulnerability to mental illness if conditions be adverse (Belsky and Pluess, 2009; Branchi, 2011). The involvement of serotoninergic mechanisms in mediating sensitivity to the environment is supported by gene-environment interaction studies that have linked certain serotonin genotypes to greater susceptibility to stress (Caspi et al., 2003). Particular focus has been placed on a serotonin transporter (5-HTT) gene polymorphism, and the finding that the (s/s) allele, associated with lower re-uptake and thus higher synaptic 5-HT (Lesch et al., 1996) is associated with a greater likelihood of depressive symptoms in response to stress (Caspi et al., 2003; Karg et al., 2011; Zammit and Owen, 2006) – although see (Mirkovic et al., 2016). Evidence of increased plasticity with SSRIs and increased 5-HT transmission more generally (Mattson et al., 2004) has been used to endorse a view that serotonin mediates susceptibility to the environment (Branchi, 2011) but little has been written about how specific 5-HT receptors mediate this effect.

Crucially, 5-HT2AR polymorphisms have been associated with: (1) increased sensitivity to stressful *and* enriching environments (Dressler et al., 2016; Fiocco et al., 2007;Jiang et al., 2016; Jokela et al., 2007; Lebe et al., 2013; Salo et al., 2011); (2) early life stress and maternal deprivation increases the availability of 5-HT2ARs and their sensitivity to excitation (Benekareddy et al., 2010; Vazquez et al., 2000); (3) time-dependent sensitisation stress which models post-traumatic stress disorder (PTSD) in rodents, increases 5-HT2AR affinity (Harvey et al., 2003); (4) chronic glucocorticoid administration to rodents increases 5-HT2AR densities (Katagiri et al., 2001); (5) 5-HT2AR availability is highest during critical development periods (Sheline et al., 2002); (6) 5-HT2AR signalling mediates behavioural responses to stress in non-human animals (Aloyo and Dave, 2007) and (7) humans experiencing psychedelic drug trips are, like children, exquisitely sensitive to their environment (Eisner, 1997; Hartogsohn, 2016), with the provision of a supportive, nurturing environment being strongly advocated for psychedelic ‘trippers’ (Johnson et al., 2008) – as for children. In summary, all these findings lend support to the notion that a key function of 5-HT2AR signalling is to mediate plasticity and associated change, especially in situations where change would be functionally advantageous.

### 5-HT2AR-mediated plasticity: an adaptive response to extreme adversity

As discussed earlier (Section 3.2.2), anxiety and stress are potent non-pharmacological inducers of 5-HT release (Bland et al., 2003b; Fujino et al., 2002; Rex et al., 2005). Anxiety and stress are most intensely evoked when survival is threatened, and accordingly, massive 5-HT release (~250 fold vs. baseline) has been detected in the rodent brain during asphyxiation and cardiac arrest, and although other neurotransmitters also show a marked increase, the increase in 5-HT release was especially marked (Li et al., 2015). It has been speculated that elevated levels of the endogenous 5-HT2AR agonist psychedelic DMT (Barker et al., 2012) may account for spontaneously occurring psychedelic-like states such as occur in near-death experiences (Strassman, 2000) but to our knowledge, empirical evidence for this theory has yet to published. Functionally, there is no more extreme condition than being proximal to death (if still fully alert). It is intriguing to consider what role may be served by enhanced serotonergic functioning during the perceived threat of death, and particularly 5-HT2AR signalling.

Indeed, similarities between the phenomenology of near-death experiences and the psychedelic state (e.g. disturbed time perception, reliving/autobiographical memory recollection, sudden insight, a sense of peace, a sense of interconnectedness and unity, a sense of other-worldliness, religious and/or mystical-type feelings – which may include a sense of presence or an encounter with (a perceived) person or deity of significance, and a message or instruction (Greyson, 2008; Greyson, 1993; Greyson and Bush, 1992; Greyson, 1983)) may rest on similarities in their pharmacology – i.e. extreme 5-HT2AR signalling. Similarly, the incipient phase of a psychosis – which may be associated with a ‘psychedelic-like’ phenomenology (e.g. a fragmenting self/ego, muddled thinking, bizarre thought content, de-realisation, mystical-type experiences and/or religious conversation or epiphany, putative insight, magical thinking, perceptual disturbances and a sense of dread etc. (Bowers and Freedman, 1966; Chapman, 1966)) may also involve exceptionally high 5-HT2AR transmission (Gouzoulis-Mayfrank et al., 1998, 2005;Gouzoulis et al., 1994).

Consistent with the bipartite model we present here (Figure 3), a stress-induced upregulation of 5-HT2AR signalling as an adaptive response to extreme adversity (Benekareddy et al., 2010; Berton et al., 1998; Harvey et al., 2003; Katagiri et al., 2001), could be an unacknowledged factor in the pathogenesis of psychosis (Gouzoulis-Mayfrank et al., 1998; Gouzoulis et al., 1994; Holloway et al., 2013). Indeed, if this hypothesis was to hold up to empirical testing, it would have important implications for how we understand and perhaps treat psychosis. For example, it could: (1) imply a role for 5-HT1AR agonism and 5-HT2AR antagonism in the moderation of the ‘prodromal state’, and/or (2) endorse the importance of providing a highly supportive environment for the at-risk individual – as is provided for actual drug-induced psychedelic experiences (Johnson et al., 2008).

Earlier (Section 3.2.2), we discussed the hypothesis that 5-HT mediates passive coping (or an improved ability to tolerate stress) under adverse conditions via postsynaptic 5-HT1AR signalling. Enhanced coping via a moderation of stress may be advantageous during difficult conditions but it may also be counterproductive, e.g. if it promotes a ready acceptance of these conditions and a compromised ability to learn from or strive to change them. Conversely, if learning and adaptability are enhanced (e.g. via 5-HT2AR signalling), then this may confer significant evolutionary advantages. Moreover, humans’ remarkable adaptability is one of our defining traits – being fundamental to our development and thriving as a species (Anton et al., 2014; Stini, 1975).

We propose that an enhanced ability to tolerate stress, mediated by enhanced postsynaptic 5-HT1AR signalling, may be a logical, adaptive response to moderate levels of adversity but that enhanced adaptability and capacity for change (e.g. in outlook and behaviour) mediated by 5-HT2AR signalling may be optimal when the level of adversity reaches a critical point – e.g. when one’s life is in danger. A number of different experiments could be performed to test this hypothesis, with its basic tenets being: (1) extremely adverse conditions evoke enhanced 5-HT2AR signalling, plasticity and propensity for change; (2) 5-HT1AR signalling dominates serotonin functioning during normal conditions and during mild-moderate adversity but 5-HT2AR plays an increasingly prominent role as the level of adversity increases to a critical point, and concentrations of synaptic 5-HT do similarly (e.g. as can be achieved experimentally with extreme stress (Amat et al., 2005), or with raphe stimulation (Amargos-Bosch et al., 2004; Puig et al., 2005)).

The rapid and transient downregulation of hippocampal 5-HT1ARs seen with severe acute stress may be one mechanism by which this hypothesised transition to 5-HT2AR dominance under extreme adversity occurs (Berton et al., 1998; Lopez et al., 1999), and increased limbic 5-HT release with mild stress (Bekris et al., 2005) compared with cortical 5-HT release with more intense stress (Amat et al., 2005) may be another. Another possibility is that 5-HT2ARs switch from their default low-affinity state to a high-affinity state (Glennon et al., 1988) under conditions of extreme stress. Indeed, this may explain why 5-HT2AR density (Katagiri et al., 2001) and affinity (Harvey et al., 2003) are increased under extreme stress in rodents. The development of 5-HT2AR agonist radioligands (Ettrup et al., 2014, 2016; Jorgensen et al., 2016) may help us to better test for altered 5-HT2AR availability in the human brain during extreme stress and to correlate this with state and trait psychological variables. Our firm hypothesis would be that 5-HT2AR binding would be significantly increased in highly stressed individuals and that this may relate to psychological and neurobiological measures of plasticity.

### Serotonin and positive mood

According to the central hypothesis of this paper, the principal function of brain serotonin is to facilitate adaptive responses to adverse conditions via two distinct pathways. Consequently, like much of the literature on the function of brain 5-HT, this paper has concentrated on adversity. This approach can be defended on a number of grounds: (1) there are plenty of relevant data on adversity because it is relatively easy to experimentally induce; (2) adverse conditions provide models from which to test experimental treatments; (3) adversity and its behavioural and biological corollaries are of central relevance to medicine and psychiatry; and, perhaps most critically, (4) negotiating adversity is of fundamental evolutionary importance.

Regarding the pharmacology of positive mood, the reliability with which potent 5-HT releasers such as MDMA and mephedrone induce marked positive mood (Carhart-Harris et al., 2011, 2015b) could be seen as supportive of the (albeit disputed (Andrews et al., 2015)) association between enhanced serotonergic functioning and positive mood. The euphoria associated with these compounds is distinct from that associated with other stimulants that have more pronounced catecholamineric releasing effects – such as methamphetamine (Bedi et al., 2014). It is intriguing to consider how much of a contribution 5-HT2AR agonism makes to the euphoric effects of MDMA and mephedrone (Schmid et al., 2015), particularly since 5-HT2AR antagonism significantly attenuates the positive mood effects of MDMA (van Wel et al., 2012) and 5-HT1AR antagonism does this less reliably (van Wel et al., 2012). PET imaging work utilising potent 5-HT releasers and receptor-selective ligands sensitive to this release (Paterson et al., 2010, 2013; Tyacke and Nutt, 2015) may be able to shed new light on the association between enhanced 5-HT transmission and positive mood (Jorgensen et al., 2016) that may help to disambiguate this matter (Andrews et al., 2015).

It would also be relevant to better understand why more selective 5-HT releasers such as fenfluramine do not produce the same kind of euphoria associated with MDMA and mephedrone, e.g. increased anxiety and decreased positive mood were seen with high doses of fenfluramine (Brauer et al., 1996), although reduced anxiety has also been observed with lower doses (Hetem et al., 1996). The contribution of catecholamine release to the MDMA and mephedrone ‘high’ may be an important factor, as may the remarkable potency of their 5-HT release, which is comparatively much greater for MDMA and mephedrone (Golembiowska, et al., 2016) than for fenfluramine (Zolkowska et al., 2008). It is also possible that the pharmacology of fenfluramine’s metabolite, norfenfluramine, which is different to that of its parent compound, e.g. norfenfluramine has greater 5-HT2C receptor agonism (Miller, 2005), may account for some of its aversive effects. Relatedly, it is known that the 5-HT2C receptor agonist mCPP tends to induce anxiety and panic (Wood, 2003).

It is important to state that 5-HT2AR agonist psychedelics are not hedonic drugs in the classic sense (Carhart-Harris and Nutt, 2013). Psychedelics are not habit forming in animals or humans (Bogenschutz and Johnson, 2016) and typical patterns of use are relatively sporadic, with protracted periods of abstinence (Nichols, 2004). However, very low (‘micro-doses’) are reportedly being taken regularly for (putative) mood and cognition enhancement (Gregoire, 2016; Waldman, 2017) and states of extreme positive mood are not infrequently reported with larger doses of psychedelics (Schmid et al., 2015), particularly when taken in supportive environments (Studerus et al., 2012) – although marked anxiety and/or dysphoria can also occur (Carbonaro et al., 2016). As highlighted in our EP model (Figure 2), *context* is likely to play an important role in determining the quality of a psychedelic experience (Hartogsohn, 2016; Roseman et al., 2017a) – and positive mood associated with 5-HT2AR agonist psychedelics may have much to do with positive expectations and environmental factors.

This said, it is intriguing to consider the possibility that a ‘loosened mind’, whether subsequent to enhanced 5-HT2AR signalling or not, may be a non-negligible component of the neurobiology of positive mood itself (Ashby et al., 1999). Blocking the 5-HT2AR has been found to significantly attenuate the positive mood effects of three different classic psychedelics (Kometer et al., 2012; Preller, 2016; Valle et al., 2016) and MDMA (van Wel et al., 2012), and it is intriguing to consider whether 5-HT2AR-mediated plasticity may be an underappreciated component of the antidepressant action of SSRIs (Chamberlain et al., 2006; Petit et al., 2014; Qesseveur et al., 2016). Several studies have demonstrated a relationship between positive mood and creative thinking (De Dreu et al., 2008; Hirt et al., 2008), with the elation, flight of ideas and general hyper-creativity of manic states being relevant in this context (Jamison, 1994).

*‘The secret to happiness is freedom’.* (Thucydides c. 450BC)

It is presumed that the brain (like the mind) functions in a freer, less constrained manner during creative states, as during positive mood (Martindale, 2007) – although this hypothesis needs to be better tested (although see Atasoy et al., 2017) – and imaging studies with potent serotonergic compounds may help in this regard (Carhart-Harris et al., 2012a, 2014d, 2015b, 2016d; Heifets and Malenka, 2016; Roseman et al., 2014). It is commonplace to refer to depressive states as excessively rigid (Holtzheimer and Mayberg, 2011); being characterised by emotional withdrawal and anhedonia, and impaired and pessimistically biased cognition (Berman et al., 2011; Holtzheimer and Mayberg, 2011), whereas the psychedelic experience is often described as psychologically *liberated* (Turton et al., 2014; Watts et al., 2017) and functional neuroimaging findings support such a description (e.g. Petri et al., 2014). A recent qualitative analysis of treatment responses to psilocybin for depression suggested that successful treatment response is characterised by a sense of having been psychologically ‘reset’, with renewed feelings of ‘connection’ and emotional ‘acceptance’ post-treatment (Roseman et al., 2017b; Watts et al., 2017). Moreover, pre- versus post-treatment fMRI data from our psilocybin for treatment-resistant depression trial suggest a potential neurobiological counterpart to the psychological notion of ‘reset’ (Carhart-Harris et al., 2017b).

## Limitations

It is appropriate to acknowledge some of limitations of this review. Only two serotonin receptor subtypes have been discussed in depth and it would be wrong to dismiss the contribution of the others. For example, some relatively new antidepressants have an important (antagonist) action at 5-HT2C receptors (which has secondary faciliatory effects on DA transmission) (MacIsaac et al., 2014) and others, such as vortioxetine, have appreciable affinities for several other 5-HT receptors (Riga et al., 2016; Thase et al., 2016) – perhaps most notably, the 5-HT6 receptor (Karila et al., 2015)). Similarly, we did not address literature on functional selectivity or agonist trafficking (Berg et al., 1998; Gray and Roth, 2001; Meana, 2013) and neither have we discussed the role of heterodimers in serotonergic and particularly 5-HT2AR functioning (Gonzalez-Maeso, 2011, 2014; Gonzalez-Maeso and Sealfon, 2012), nor the role of glutamatergic mechanisms that follow 5-HT2AR signalling and how these are involved in plasticity (Aghajanian and Marek, 1999). It should also be acknowledged that much importance has been ascribed to psychedelics’ 5-HT2AR agonist properties but many of the psychedelic compounds featured also possess considerable actions at other 5-HT receptors, including the 5-HT1AR (Nichols, 2004). Although we acknowledge this limitation, we also wish to emphasise that the evidence is compelling that 5-HT2AR agonism is *key* to psychedelics’ most characteristic effects (Halberstadt, 2015), 5-HT1AR agonism attenuates rather than augments these effects (Pokorny et al., 2016; Strassman, 1996) and more selective 5-HT2AR agonists appear to have the same quintessential psychological effects as the less selective psychedelics (Halberstadt, 2017).

We acknowledge that what is presented here is a simplified and therefore incomplete picture of brain serotonin function. This was an intentional approach (and compromise) however, as our main aim was not to produce an exhaustive review of serotonin transmission at its many receptors but rather distil it down to some key principles. We chose to focus on the 5-HT1A and 2A receptors because we felt that the functions associated with their signalling give the most comprehensive perspective of the general functioning of brain serotonin transmission. These two receptors are more implicated in the pharmacology of major psychiatric disorders than any of the other 5-HT receptor subtypes (Artigas et al., 2013b; Azmitia, 2007) – although others have highlighted the 5-HT1B receptor using a similar argument (Nautiyal and Hen, 2017) and it must be conceded that *wealth of data* does not necessarily imply *strength of relationship*. However, that the 5-HT1A and 2A receptors have opposite effects on single cell activity has long been a matter of intrigue (Araneda and Andrade, 1991). Crucially, that these receptors also seem to subserve distinct functions (Table 1) implies that the 5-HT system is not just diverse, but *adaptive*. We propose that the 5-HT system is specifically adaptive to the severity of adversity and whether it is better to passively tolerate it (with the assistance of 5-HT1AR signalling) or more actively respond it via a major change in perspective and/or behaviour (with the assistance of 5-HT2AR signalling).

Another criticism of this paper is that it has focused too much on 5-HT2AR agonist psychedelics and MDMA, rather than on classical preclinical behavioural literature and less potent serotonergic manipulations. In defence of our approach, the primacy we have given to research on psychedelics has allowed us to conceive a truly novel model of brain serotonin function. The most unique component of our model is pathway 2 (Figure 3), i.e. that 5-HT2AR signalling mediates plasticity related processes in aid of active coping. That this pathway has not previously been emphasised in models of serotonin function may have been due to a historical focus on the association between 5-HT2AR agonism and pathology and an insufficient willingness to acknowledge and endeavour to study these drugs’ complex subjective effects. We share the view of others (Grof, 1979; Heifets and Malenka, 2016) that 5-HT2AR agonist psychedelics and MDMA are remarkably powerful tools for studying the human brain and mind – and their scientific and medicinal value has not yet been properly appreciated (Carhart-Harris and Goodwin, 2017). We also believe that human studies with these compounds can be done safely if appropriate safeguards are heeded (Johnson et al., 2008).

It could be argued that too much emphasis has been placed on extreme states in this paper that are not relevant to normal physiological conditions. Basal 5-HT2AR signalling has shown to be important for the maintenance of normal levels of cognitive flexibility (Boulougouris et al., 2008; Clarke et al., 2004, 2007) and may also account for traits such as high ‘absorption’ (Ott et al., 2005). We subscribe to the principle that challenging a system with an extreme perturbation can yield especially valuable insights about its normal functioning, by pushing it to and beyond its limits. Moreover, given that evolutionary pressures are major drivers of adaptation and change, understanding how a particular function operates during extreme conditions (e.g. when one’s life is in danger), may be particularly informative about why that function exists at all. It seems reasonable to infer that states induced by MDMA and 5-HT2AR agonist psychedelics may be possible to achieve without these drugs, if only at an attenuated level. These drugs may therefore justifiably be considered ‘unveilers of function’. Note: the term ‘psychedelic’ literally means ‘mind-revealing’.

Relatedly, it is intriguing to speculate that 5-HT2AR signalling may have played an important role in human evolutionary as well as ontogenetic development, perhaps through enhancing plasticity and adaptability during extreme conditions. The 5-HT2AR is densest in evolutionary recent brain regions (Beliveau et al., 2016; Erritzoe et al., 2009; Ettrup et al., 2014, 2016; Varnas et al., 2004; ). Indeed, it is readily apparent in Figure 1 that 5-HT2AR expression is especially dense in regions of the so-called default-mode network, which is associated with especially high-level psychological functions, such as self-consciousness and the ‘self’ or ‘ego’ itself (Carhart-Harris and Friston, 2010) as well as the acute network level effects of psychedelics, as determined by human neuroimaging studies (Carhart-Harris et al., 2014). By body weight, humans have vastly more cortex than other species (MacLean, 1990; Molnar et al., 2014) (where 5-HT2ARs are densest (Ettrup et al., 2014)) and our remarkable adaptability is one of our most defining species traits (Anton et al., 2014) – as is our sense of self. It has been hypothesised by a popular proponent of psychedelic drug-use (Terrence McKenna) that ingestion of naturally occurring psychedelics (e.g. psilocybe mushrooms) catalysed the evolution of the human neocortex (Abraham et al., 1998). A perhaps more plausible (and less psychedelic-centric) alternative however, is that non-linearities evolved in the serotonergic system (Erritzoe et al., 2010; Jansson et al., 2001) that conferred optimal adaptability, including a capacity to switch to greater 5-HT2AR signalling when conditions demand it (such as during extreme adversity). Future work may endeavour to test the hypothesis that 5-HT2AR signalling serves an exceptional function in humans. The vastness of our 5-HT2AR dense cortex suggests that this hypothesis is worth exploring, and the development of agonist radioligands that can label the 5-HT2AR in its high affinity state may help us in this regard (Ettrup et al., 2014, 2016; Jorgensen et al., 2016).

Regarding neuroimaging the psychedelic state, this is a nascent and fast-moving field and it would be beyond the scope of this article to discuss the relevant published findings in detail (this area is deserving of its own review paper). Suffice to say that an emergent principle from the various studies is that the brain is uncharacteristically ‘entropic’ in the psychedelic state (Carhart-Harris et al., 2014), reflecting a greatly heightened plasticity in which old material may be unlearned (consistent with the principles of extinction learning) and new ideas and associations learned.

It might be argued (unfairly in our view) that the present contribution on the function of brain serotonin has not added anything new to previous models (Andrews et al., 2015; Azmitia, 2007; Branchi, 2011; Dayan and Huys, 2009; Deakin, 1998). We acknowledge that the model presented here has been much inspired by previous attempts to resolve this enigma but feel it also significantly advances on them and is entirely novel in its own right. It integrates findings that were inspirational for previous models but also assimilates recent and (perhaps somewhat overlooked) data on the brain and behavioural effects of potent serotonergic drugs such as MDMA and the 5-HT2AR agonist psychedelics. Previous models acknowledged the role of hippocampal 5-HT1AR signalling in resilience (Deakin, 2013; Deakin and Graeff, 1991) but we have significantly extended on this by our thorough coverage of 5-HT2AR functioning and its mediation of plasticity in aid of optimal adaptability.

Regarding specific past contributions, we acknowledge the work of Deakin and Graeff (Deakin, 2013; Deakin and Graeff, 1991) and others (Cools et al., 2008; Crockett et al., 2009; Wise et al., 1970) concerning the role of 5-HT in aversive processing, plus the increasingly compelling work on serotonin’s role in promoting patience (Fonseca et al., 2015; Miyazaki et al., 2012, 2014) and collectively relate these to our hypothesis that postsynaptic 5-HT1AR signalling mediates passive coping in response to adversity. It is worth commenting on a nuance here: in Deakin and Graeff’s model, 5-HT1AR signalling is linked to *chronic* adversity – which we do not dispute; however, we would argue that 5-HT2AR signalling becomes increasingly relevant as the *severity* of adversity reaches a critical point. Indeed, we have emphasised the importance of the severity of adversity in our model – but it may be worthwhile to also consider the role of the *chronicity* of adversity in determining the differential engagement of 5-HT receptor subtypes (Cohen et al., 2015; Dayan and Huys, 2009).

We also acknowledge the increasingly appealing perspectives of Branchi (2011), Belsky et al. (2009) and others (Homberg, 2012) concerning serotonin and plasticity, and relate this to our hypothesis that 5-HT2AR signalling mediates plasticity in aid of optimal adaptability. We acknowledge Andrew et al.’s hypothesis of serotonin mediating an adaptive homeostasis (Andrews et al., 2015) (see also Hale et al. (2013)) and believe this could be broadly related to our bipartite model. However, we feel our model is more psychologically focused, receptor specific, and consistent with the classical view that enhanced 5-HT transmission (within certain bounds and contexts) is conducive to positive mood. Perspectives such as Andrews and colleagues (2015) that challenge this view, cite, among other things, the relationship between punishment, 5-HT release and depression – to endorse the perspective that serotonergic functioning is elevated in depression (Barton et al., 2008)). Consistent with the classical (Wise et al., 1970) and arguably still dominant perspective (Cowen and Browning, 2015) however, our view is that increased 5-HT release in response to adversity is *functional* rather than pathological, serving to moderate stress via postsynaptic 5-HT1AR signalling, and in extreme cases, initiate a rapid plasticity in the service of major change – via 5-HT2AR signalling.

## Conclusions: the function of brain serotonin

This paper has sought to address a major unresolved problem in neuropsychopharmacology, namely *what is the function of brain serotonin?* It proposes that the principal function of brain serotonin is to enhance adaptive responses to adverse conditions via two distinct pathways: (1) a passive coping pathway which improves stress tolerability; and (2) an active coping pathway associated with heightened plasticity, which, with support, can improve an organism’s ability to identify and overcome source(s) of stress by changing outlook and/or behaviour. Crucially, we propose that these two functions are mediated by signalling at postsynaptic 5-HT1A and 5-HT2A receptors respectively, with 5-HT1AR signalling dominating under ordinary conditions but 5-HT2AR signalling becoming increasingly operative as the level of adversity reaches a critical point.

We suggest that the two functions of interest (5-HT1AR-mediated stress relief and 5-HT2AR-mediated plasticity) are sufficiently distinct – and may even be mutually oppositional in certain contexts (see also Azmitia, 2001), evoking dilemmas over whether it is better to passively endure or actively approach, and in so doing, initiate some sort of fundamental change – with the potential for major resolution. This rule may not be absolute however, and the two functions may also be complementary, e.g. in the case of enhanced serotonin functioning with chronic SSRI use – or indeed with normal basal 5-HT functioning, facilitating improved endurance *and* plasticity (Clarke et al., 2004, 2007; Mithoefer et al., 2011, 2016; van Apeldoorn et al., 2008).

Despite this complementarity, we do anticipate that conventional serotonergic antidepressants such as the SSRIs and classic psychedelics such as psilocybin may become *competitive* options for the treatments of certain disorders such as depression; most fundamentally because they work via distinct pathways (i.e. 5-HT1AR versus the 5-HT2AR signalling) – but also because they cannot easily be taken in combination, i.e. conventional antidepressants attenuate the characteristic psychological effects of psychedelics (Bonson et al., 1996; Bonson and Murphy, 1996). SSRIs are established evidence-based treatments for anxiety and major depression (Baldwin et al., 2016; Hieronymus et al., 2016), whereas psychedelics are experimental medicines in an early phase of development (Carhart-Harris and Goodwin, 2017; Carhart-Harris et al., 2016). However, if evidence supporting the therapeutic value of psychedelics accrues – as we anticipate, and it is increasingly shown that their therapeutic mechanisms are significantly distinct from those of conventional medications, then this will open-up new and potentially empowering options for patients and clinicians (as well as a real potential for resistance – however it may arise). For the brave new psychiatry of the future – that many would like to see (Miller, 2010) – decisions about whether to *passively endure* or *actively address*, may become increasingly pertinent.

‘*Progress is impossible without change, and those who cannot change their minds cannot change anything*’. (George Bernard Shaw)
